# Indel pattern-guided repair mapping reveals genome-wide DNA repair networks in CRISPR/Cas9 editing

**DOI:** 10.1093/nar/gkag260

**Published:** 2026-03-27

**Authors:** Yanbin Wan, Xuanye Zhao, Xiding Lin, Lv Wang, Xiaoqiang Ai, Jianmin Jiang, Liya Han, Dongchao Huang, Hongli Du, Lizhen Huang

**Affiliations:** School of Biology and Biological Engineering, South China University of Technology, Guangzhou 510006, China; School of Biology and Biological Engineering, South China University of Technology, Guangzhou 510006, China; School of Biology and Biological Engineering, South China University of Technology, Guangzhou 510006, China; School of Biology and Biological Engineering, South China University of Technology, Guangzhou 510006, China; School of Biology and Biological Engineering, South China University of Technology, Guangzhou 510006, China; School of Biology and Biological Engineering, South China University of Technology, Guangzhou 510006, China; School of Biology and Biological Engineering, South China University of Technology, Guangzhou 510006, China; Medical Research Institute, Wuhan University, Wuhan 430071, China; School of Biology and Biological Engineering, South China University of Technology, Guangzhou 510006, China; FangRui Institute of Pharmaceutical Innovation, South China University of Technology, Guangzhou 510006, China; School of Biology and Biological Engineering, South China University of Technology, Guangzhou 510006, China; FangRui Institute of Pharmaceutical Innovation, South China University of Technology, Guangzhou 510006, China

## Abstract

CRISPR/Cas9-induced DNA double-strand breaks (DSBs) trigger diverse repair outcomes, yet the dynamic regulatory networks governing these outcomes remain incompletely understood. Here, we develop indel pattern-guided repair mapping, an integrative framework that deciphers DSB repair mechanisms by integrating repair outcome spectra, kinetic dynamics, and functional gene regulation. Our analysis categorizes Cas9-mediated repair outcomes into seven distinct patterns based on their frequency and sequence characteristics, revealing differential repair kinetics among these subtypes. Functional clustering identifies three regulatory pillars: (i) microhomology-mediated end joining (MMEJ)-driven MH deletions form a cohesive module defined by a shared regulatory network of protein-coding genes and miRNAs, rather than by the core repair enzymes themselves; (ii) non-homologous end joining coordinates 1 bp insertions and non-MH deletions, with RFC4/5 stabilizing repair templates to suppress large deletions; (iii) Atypical repair outcomes show distinct genetic signatures: large insertions are associated with polymerase-related regulators, whereas mutations are associated with a signature enriched for chromatin-associated regulators. Strikingly, S100A8 emerges as a potent MMEJ suppressor via direct interaction with PARP1, revealing unappreciated cross-talk between inflammatory signaling and DSB repair pathway choice. By linking repair outcome patterns to molecular determinants, our work provides a transformative platform to interrogate DNA repair mechanisms for precise genome editing optimization and therapeutic genome stabilization.

## Graphical Abstract

**Graphical Abstract:**
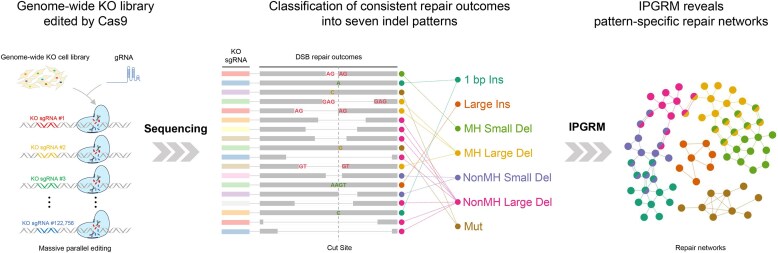

## Introduction

Programmable CRISPR/Cas technology has revolutionized genome editing, serving as a cornerstone for both fundamental research and therapeutic development. The efficacy and precision of CRISPR-based editors—ranging from Cas9-mediated knockout to base editing (BE) and prime editing (PE)—are fundamentally governed by cellular DNA repair pathways. Cas9 generates double-strand breaks (DSBs), engaging non-homologous end joining (NHEJ) or homology-directed repair (HDR) to dictate editing outcomes such as insertions, deletions, or precise replacements. Meanwhile, BE and PE introduce single-strand breaks (SSBs) or base lesions, engaging mismatch repair (MMR) or translesion synthesis to shape final edits [1]. The diversity of repair mechanisms underscores a critical challenge: achieving predictable editing requires unraveling the complex interplay between DNA damage types and their corresponding repair networks [2].

To dissect repair pathways genome-wide, Repair-seq has emerged as a powerful tool, linking repair genes to specific editing outcomes at single-base resolution [3]. Unlike traditional reporter assays limited to predefined repair phenotypes [4–6], Repair-seq concurrently profiles thousands of repair outcomes and their genetic dependencies through deep sequencing [3, 7, 8]. However, scaling Repair-seq to genome-wide screens presents technical hurdles: (i) ultra-high sequencing depth is required to resolve repair outcomes across >20 000 genes, limiting library complexity [3, 7]; (ii) redundant gene enrichments arise due to stochastic or sequence-context-dependent repair outcomes [9], obscuring direct causal relationships. These constraints highlight the need for innovative approaches to map repair networks efficiently.

Notably, Cas9-induced DSB repair exhibits striking patterns despite its apparent randomness. Genome-wide analyses reveal recurrent indel signatures, such as predominant 1-bp insertions, consistent with the fact that Cas9 generates heterogeneous DSB end structures—predominantly blunt ends but also a substantial, sequence-context–dependent fraction of 5′ overhangs that can be filled in prior to end joining, or microhomology-mediated deletions [10–12]. Such predictability has fueled the development of computational tools (e.g. inDelphi, FORECasT, SPROUT) to forecast editing outcomes based on sequence context [13]. These observations suggest that indel patterns are not stochastic but reflect underlying repair pathway preferences—a paradigm we leverage to refine repair gene discovery.

Here, we perform high-throughput sequencing of Cas9-induced indels across diverse chromosomal loci, identifying seven recurrent outcome classes defined by indel type (insertion/deletion), length, microhomology usage, and kinetics. These patterns serve as a roadmap to develop indel pattern-guided repair mapping (IPGRM), a method coupling indel signatures with focused genetic screens to systematically identify repair factors. IPGRM bypasses the limitations of conventional Repair-seq by targeting mechanistically informative outcome classes, enabling genome-wide repair network elucidation with reduced noise and higher resolution. Our work not only expands the catalog of DSB repair regulators but also provides a framework to optimize precision editing tools by harnessing endogenous repair biases.

## Materials and methods

### Cell culture

HEK293T cells (Stem Cell Bank, Chinese Academy of Sciences, Shanghai, China) were cultured in Dulbecco’s modified Eagle medium supplemented with 10% fetal bovine serum, 100 U/ml penicillin, and 100 µg/ml streptomycin at 37°C with 5% CO₂.

### Repair outcome profiling across diverse chromatin contexts target sites

To characterize Cas9-induced DSB repair outcomes, seven sgRNAs targeting genomic loci within distinct chromatin contexts were designed and synthesized [14]. Details of the oligonucleotides are provided in Supplementary Table S1. Cas9/sgRNA ribonucleoprotein (RNP) complexes were assembled at a 1:1.2 molar ratio. HEK293T cells were electroporated using the Neon Transfection System (1 200 V, 20 ms, 2 pulses) and harvested after 48 h. Genomic DNA was extracted using MiniBEST Universal Genomic DNA Extraction Kit (TaKaRa). Polymerase chain reaction (PCR) amplifications were performed in 20 μl reactions containing 1 μg genomic DNA, 0.4 μM of each primer, and Q5 Hot Start HiFi PCR Master Mix (NEB). Cycling conditions were as follows: 98°C for 2 min; 20 cycles of 98°C for 10 s and 65°C for 45 s; and a final 5 min extension at 65°C. PCR products were purified using Cycle Pure Kit (Omega) and size-selected by Gel Extraction Kit (Omega). Purified amplicons were submitted to GENEWIZ for paired-end 150 bp (PE150) sequencing, generating 5 Gb of data per sample. Sequencing reads were processed with CRISPResso2 (v2.1.3) to quantify insertions, deletions, and substitutions.

### Establishment of repair outcome classification

Indel patterns identified from the seven-locus analysis were used to define a standardized classification framework based on sequence features such as indel length and microhomology usage. This classification system was subsequently applied to all kinetic modeling and genome-wide IPGRM analyses.

### Analysis of temporal dynamics of Cas9-induced indel patterns

Six sgRNAs located within distinct chromatin environments were selected. Cells were harvested at 0, 3, 6, 12, 18, 24, 36, 48, 60, and 72 h post-electroporation. Genomic DNA was extracted, amplified, and sequenced as described earlier for repair outcome profiling. Barcoded primers were used to distinguish time points (Supplementary Table S1), and sequencing reads were demultiplexed using fastq-multx (v1.4.2), followed by processing with CRISPResso2 (v2.1.3). The proportions of seven indel patterns were quantified at each time point to generate time-resolved outcome profiles.

An ordinary differential equation model, adapted from Brinkman *et al.* [15], was constructed to fit the temporal data and estimate kinetic parameters for DSB cleavage and subsequent repair. Briefly, we first fit the observed editing fraction over time with a logistic curve to estimate the maximal editable fraction for each target. We then fit the ODE model by least squares, fixing the non-edited fraction based on the logistic fit, and evaluated model performance using *R*^2^ and residual patterns over time. Rate constants derived from model fitting were first-order, per-target-locus parameters within the editable cell fraction, and were used to compare pathway kinetics across loci and to assess conserved temporal behaviors among distinct chromatin contexts.

### Construction and screening of the IPGRM library

To systematically identify genetic factors influencing Cas9-induced DSB repair outcomes, IPGRM library was established. The human GeCKOv2 lentiviral library (GENEWIZ, Suzhou), containing 122 756 sgRNAs targeting 19 050 genes (6 sgRNAs per gene), 1864 miRNAs (4 sgRNAs per miRNA), and 1000 NonTarget controls, was transduced into 3 × 10⁸ HEK293T cells at an MOI of 0.3. After 24 h, cells were selected with 1 μg/ml puromycin. After selection, surviving cells were expanded for 4 days, and the resulting IPGRM cell pool was subjected to secondary editing. In brief, a total of 6.3 × 10^7^ IPGRM library cells were seeded into three T175 flasks 24 h before transfection and cultured to 70%–80% confluence. Each flask was co-transfected with 7.5 μg pCMV-Cas9 and 15 μg pU6-sgRNA plasmids targeting either sg3 or sg81 (Supplementary Table S1).

Cells were harvested 48 h post-transfection, and genomic DNA was extracted. PCR reactions were performed in 100 μl volumes containing 40 μg genomic DNA. Amplicons were purified, gel-extracted, and subjected to PE150 sequencing, yielding ~46 Gb of data per sample. The resulting reads, containing both the library sgRNA and secondary DSB site, provided a direct link between knockout identity and repair outcome profile, enabling genome-wide functional screening of DSB repair modulators.

### Data processing and sgRNA–repair mapping

Paired-end reads were merged using flash (V1.2.11; -m 10 -M 100 -x 0.25 -O), filtered with fastp (V0.23.2; -w 5 -q 20 -n 1 -l 50), and trimmed with fastq-multx (V1.4.2) to ensure consistent start positions. Subsequently, MAGeCK count (v0.5.9.4) was used to quantify sgRNA distributions and evaluate library representation, while CRISPResso2 (v2.1.3) was applied to extract repair profiles at target sites for consistency checking.

High-quality reads were first aligned to the reference sequence, while unedited reads were removed. Filtered reads were then mapped to the GeCKOv2 library file based on positional matching of sgRNA sequences, using a strict mismatch-free mode to accurately determine the corresponding sgRNA and gene identity. Edited sequences were realigned to the reference sequence using Biopython’s pairwise2 module. Sequence alterations overlapping or adjacent to the Cas9 cleavage site were classified as repair events and further categorized into specific indel patterns using the classification framework described earlier.

### Enrichment and integrative analysis of indel pattern associated genes

For each repair outcome category, sgRNA enrichment was evaluated by comparing outcome frequencies between individual sgRNAs and nontargeting controls using Fisher’s exact test. Both left-tailed (depletion) and right-tailed (enrichment) *P*-values were computed together with the corresponding log₂ fold change (log₂FC). sgRNAs with ≤10 reads or without detectable mutations were excluded.

To derive gene-level significance, sgRNAs were ranked globally based on direction-specific *P*-values and log₂FCs. For depletion analysis, sgRNAs showing negative log₂FCs and smaller left-tailed p-values were prioritized, while inconsistent entries were penalized. The same strategy was mirrored for enrichment analysis. Robust Rank Aggregation (RRA) was applied to these ranked lists to compute gene-level RRA scores [16]. Statistical significance was assessed through 20 000 random permutations to generate empirical *P*-value distributions.

To integrate results from the sg3 and sg81 libraries, shared genes were retained, and combined log₂FC values were calculated using outcome-frequency–weighted averages. Corresponding one-tailed *P*-values were transformed into *z*-scores, merged by weighted averaging, and back-transformed into integrated *P*-values. Genes with |log₂FC| > 0.5 and combined *P* < .05 were considered significantly associated with specific repair outcome categories. Details of the significantly differential genes are provided in Supplementary Table S2.

### Clustering and correlation analysis of significantly differential genes

For each of the seven indel patterns, the top 50 promoting and top 50 inhibiting genes were selected based on gene-level *P*-value rankings, resulting in a total of 700 genes for downstream analysis. Hierarchical clustering was performed on log₂FC profiles of these genes across all indel patterns using the “average” linkage method to reveal functional grouping patterns. Pearson correlation coefficients were computed between every pair of genes to assess similarity in repair outcome regulation. Heatmaps were visualized using the pheatmap and corrplot packages in R.

### Identification and network integration of candidate repair regulators

Candidate repair-associated genes and miRNAs identified through IPGRM screening were subjected to network-based integration analysis. Protein-coding genes were uploaded to the STRING database to obtain experimentally validated and predicted protein–protein interactions. The resulting network was visualized and analyzed in Cytoscape (v3.10.1), and densely connected subnetworks were identified using the MCODE plugin to reveal potential functional repair complexes. For miRNAs, target prediction was performed using TargetScan and miRDB, retaining targets identified by both databases. Functional enrichment analysis was then conducted to identify genes significantly associated with DNA damage and DSB repair pathways.

### Survival analysis

Survival analysis of candidate genes was performed using the Kaplan–Meier Plotter for breast cancer [17]. Only chemotherapy-treated patients were included, and relapse-free survival (RFS) was used as the clinical endpoint. Patients were stratified into high- and low-expression groups based on gene expression levels to evaluate survival differences. For S100A8, stratified analysis was further performed according to PARP1 expression. TCGA breast cancer datasets were downloaded, and chemotherapy-treated patients were divided into PARP1-high and PARP1-low subgroups. Kaplan–Meier analysis was then performed within each subgroup to assess the association between S100A8 expression and RFS.

### Functional and transcriptomic validation of S100A8

A stable S100A8-overexpressing HEK293T cell line was generated using a third-generation lentiviral packaging system and verified by sequencing and quantitative PCR. Both wild-type and S100A8-overexpressing cells were subjected to Cas9/sgRNA RNP-mediated editing at loci representing distinct chromatin contexts. Repair outcome distributions were analyzed following the same workflow described for the IPGRM pipeline.

To further explore transcriptional effects, RNA-seq datasets of S100a8-overexpressing and control cells were obtained from the GEO database (GSE216600) [18]. FPKM values were converted to TPM for expression comparison. Additionally, expression profiles of candidate repair-related genes were analyzed in breast cancer and normal tissues using GEPIA2.

### Statistical analysis

All statistical analyses were performed using GraphPad Prism 8. Group comparisons were conducted using two-tailed student’s t-tests, and survival curves were analyzed with the log-rank (Mantel–Cox) test. Statistical significance was defined as follows: *P < .05, **P < .01, ***P < .001, and ****P < .0001. All data are presented as mean ± standard deviation (SD) unless otherwise stated.

## Results

### Systematic classification of DSB repair outcomes reveals seven distinct indel patterns

**Figure 1.**
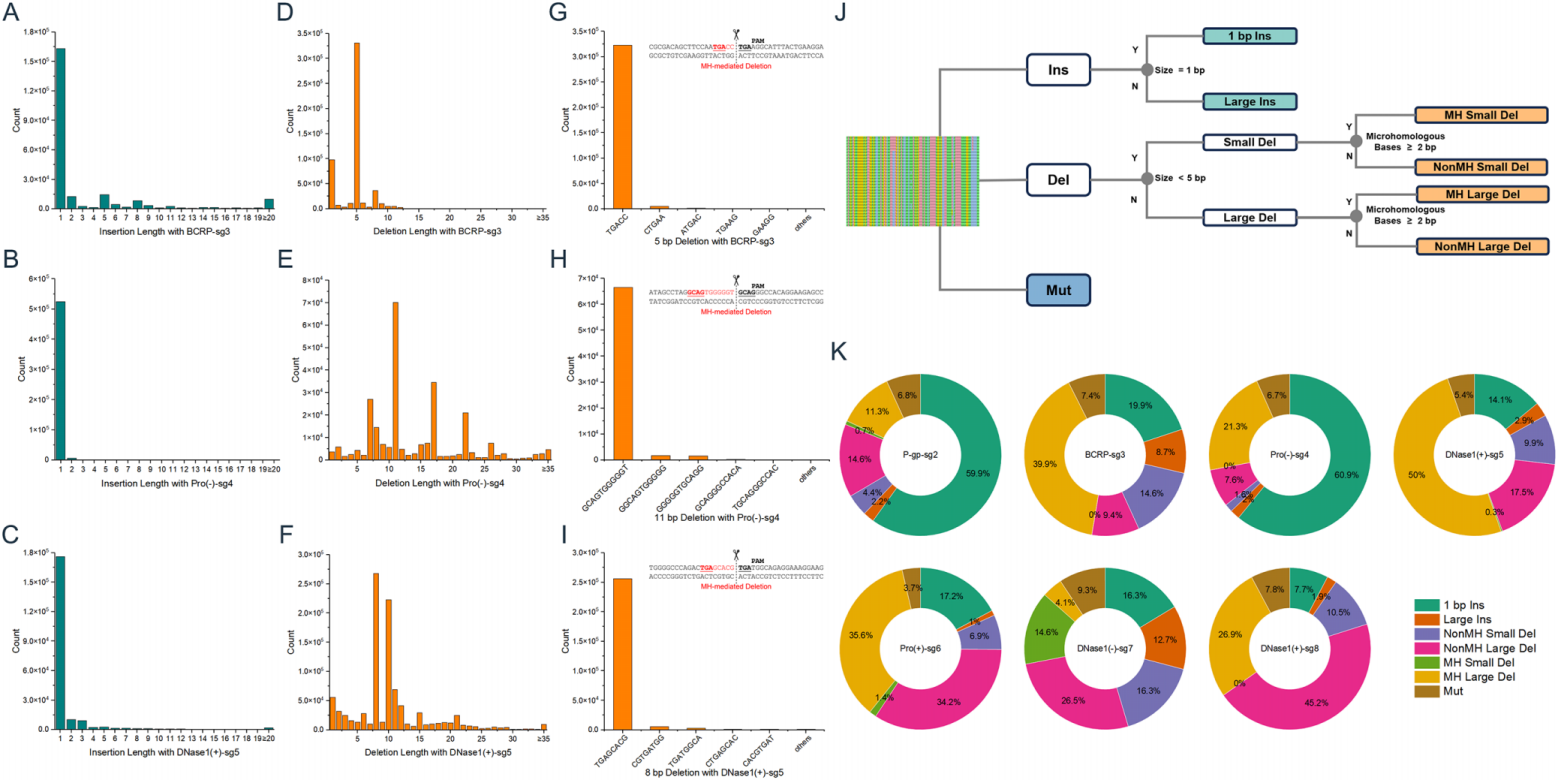
Distribution and classification of Cas9-induced repair outcomes across target loci. Insertion length distribution at BCRP-sg3 (**A**), Pro(–)-sg4 (**B**), and DNaseI(+)-sg5 (**C**). Deletion length distribution at BCRP-sg3 (**D**), Pro(–)-sg4 (**E**), and DNaseI(+)-sg5 (**F**). Sequence profiles of the most frequent deletions: 5 bp at BCRP-sg3 (**G**), 11 bp at Pro(–)-sg4 (**H**), and 8 bp at DNaseI(+)-sg5 (**I**). Microhomologous bases are indicated by underlining. (**J**) Workflow for classification of DSB repair outcomes into seven indel patterns: 1 bp Ins, Large Ins, MH Small Del, NonMH Small Del, MH Large Del, NonMH Large Del, and Mut. (**K**) Proportional distributions of the seven indel patterns across seven target sites. Abbreviations: Ins, insertion; Del, deletion; Mut, mutation; MH, microhomology.

We first characterized Cas9-induced repair outcomes across seven genomically diverse target sites by high-throughput sequencing (Fig. 1 and Supplementary Figs S1–S4). Although the relative frequencies of specific outcomes varied across loci, several consistent patterns were observed. Deletions were the predominant outcome at five targets (62%–82%) but were reduced to ∼30% at P-gp-sg2 and Pro(–)-sg4 (Fig. 1D–F and Supplementary Fig. S1E–H). Conversely, insertions dominated at P-gp-sg2 and Pro(–)-sg4 (∼60%) but were less frequent at the remaining sites (9%–29%) (Fig. 1A–C and Supplementary Fig. S1A–D). Mutations were the least frequent (3.7%–9.3%) (Supplementary Fig. S4H–N). Most insertions were 1 bp (56.2%–96.8%), with a substantial fraction templated from flanking DNA, consistent with either polymerase-mediated fill-in during repair or direct filling of short 5′ overhangs generated by Cas9 prior to end joining (Supplementary Fig. S2). Deletions exhibited greater diversity but were often mediated by 2–4 bp microhomology (MH), accounting for 30%–70% of events and dominating at certain targets (up to 97.5% of the most frequent deletion), reflecting the more defined nature of microhomology-mediated end joining (MMEJ) (Fig. 1G–I and Supplementary Fig. S1I–L). In contrast, non-MH deletions showed broader length variability, indicative of more stochastic repair. Notably, deletion breakpoints showed a consistent PAM-distal enrichment across target sites (Supplementary Fig. S3). Base substitutions clustered within 5 bp of the cleavage site, enriched on both PAM-proximal and distal sides, consistent with error-prone repair near the DSB (Supplementary Fig. S4A–G).

Based on these conserved features, we developed a comprehensive classification system that groups repair outcomes into seven mechanistic indel patterns (Fig. 1J and K): (i) 1 bp insertions (1 bp Ins); (ii) rare, large insertions (Large Ins); (iii–iv) MH-associated small (1–5 bp) and large (>5 bp) deletions; (v–vi) non-MH small and large deletions; and (vii) localized mutations (Mut). This taxonomy captures the full spectrum of repair outcomes while linking them to underlying biological mechanisms, with MH usage and indel size serving as key discriminators between major repair pathways. The consistent patterns observed across diverse chromosomal contexts indicate that, although local sequence features influence outcome frequencies, the fundamental repair principles governing Cas9-induced DSB resolution are shared genome-wide.

### Conserved temporal dynamics of DNA repair outcomes across genomic loci

**Figure 2.**
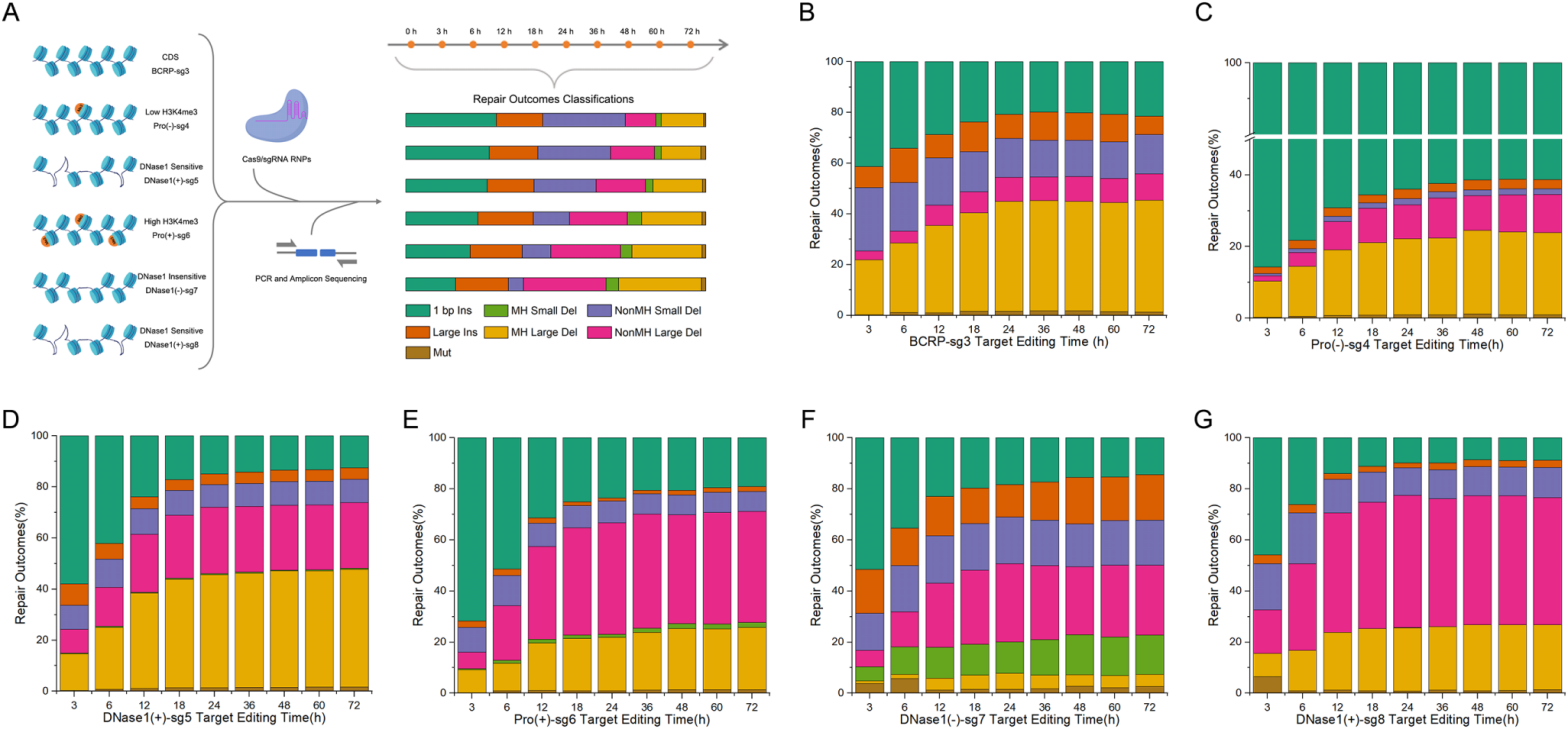
Temporal dynamics of Cas9-induced repair patterns across multiple genomic loci. (**A**) Schematic of the time-course experimental workflow. Cas9/sgRNA RNPs were electroporated into HEK293T cells targeting six genomic loci representing distinct chromatin contexts. Repair outcomes were collected at indicated timepoints by PCR amplification and amplicon sequencing, followed by classification into seven indel patterns. Details in Methods. Temporal changes in the relative proportions of repair patterns at BCRP-sg3 (**B**), Pro(–)-sg4 (**C**), DNaseI(+)-sg5 (**D**), Pro(+)-sg6 (**E**), DNaseI(–)-sg7 (**F**), and DNaseI(+)-sg8 (**G**).

Following characterization of repair features, we systematically investigated the temporal dynamics of different indel patterns across six genomic targets in various chromatin contexts (Fig. 2A). Time-course amplicon sequencing revealed that while cleavage rates were generally consistent across targets [except Pro(–)-sg4 showing higher activity], error-prone repair rates exhibited modest variation (0.0948–0.1766 h^−1^), suggesting limited but detectable influence of local sequence and chromatin environments on indel formation efficiency (Supplementary Fig. S5A–G). In contrast, perfect repair rates showed substantially greater variability (9.93 × 10^−9^–1.77 × 10^−5^ h^−1^) with overall lower values, indicating that error-free repair is both rare and more strongly dependent on chromatin context [15]. These results demonstrate that while initial DSB formation and error-prone repair proceed with relatively consistent kinetics across loci, perfect repair remains an infrequent event whose efficiency varies considerably by genomic location.

Despite these locus-specific differences in absolute rates, all six targets exhibited strikingly similar temporal patterns in the relative proportions of different indel patterns. The 1 bp Ins (Fig. 2B–G) consistently appeared first, reaching its peak within 3–6 h before gradually declining, which reflects the rapid engagement and subsequent resolution characteristic of canonical NHEJ. In contrast, Large Ins and Mut displayed either gradual accumulation or stable persistence (Fig. 2B–G), suggesting these repair processes operate over extended timescales. Most notably, MH-mediated deletions (MH Small Del and MH Large Del), along with NonMH Large Del, showed delayed emergence (Fig. 2B–G), with MH-dependent products particularly exhibiting consistent late-phase accumulation patterns that may reflect the temporally delayed nature of MMEJ pathway activation. These conserved dynamics across genomically diverse targets suggest that, while local chromatin and sequence features may influence the absolute efficiency of specific repair pathways, the fundamental timing and order of their engagement follow an intrinsic, locus-independent program.

### Development and validation of the indel pattern-guided repair mapping system

**Figure 3.**
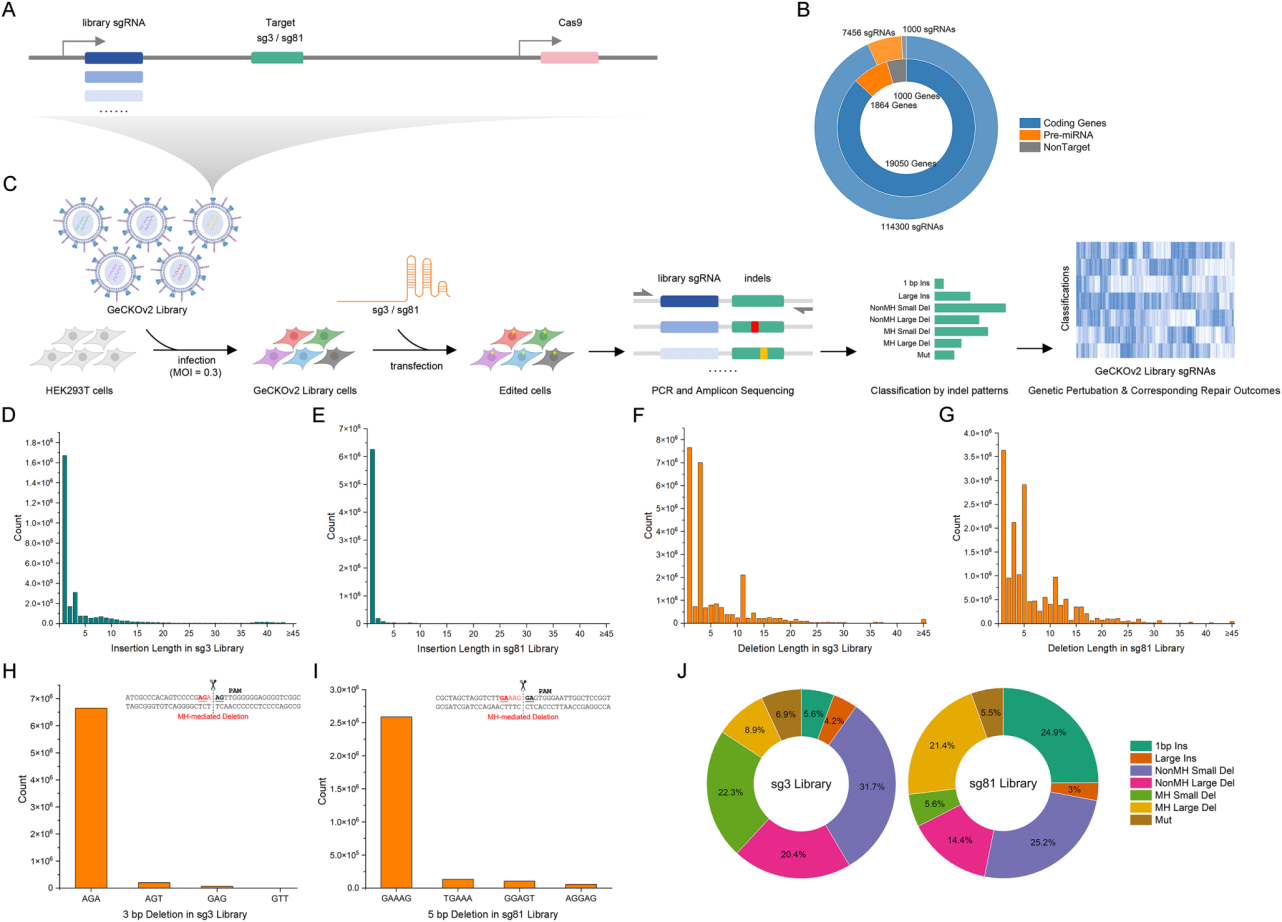
IPGRM as a genome-wide platform for systematic dissection of DSB repair mechanisms. (**A**) Schematic of vectors encoding the GeCKOv2 CRISPR knockout library with secondary Cas9 cleavage sites (sg3 or sg81) positioned adjacent to library-encoded sgRNAs. (**B**) Composition of the GeCKOv2 library, including coding genes, pre-miRNA loci, and non-targeting controls. (**C**) Workflow of IPGRM. HEK293T cells were transduced with the GeCKOv2 library and subsequently edited at sg3 or sg81 loci. Repair outcomes were captured by PCR amplicon sequencing and classified into seven indel patterns, allowing each sgRNA-mediated knockout to be directly linked to a corresponding repair outcome profile. Insertion length distributions in the sg3 (**D**) and sg81 (**E**) libraries. Deletion length distributions in the sg3 (**F**) and sg81 (**G**) libraries. Sequence profiles of the most frequent deletions: 3 bp in the sg3 library (**H**) and 5 bp in the sg81 library (**I**). Microhomologous bases are indicated by underlining. (**J**) Proportional distributions of the seven indel patterns in the sg3 and sg81 libraries.

To systematically dissect DSB repair mechanisms genome-wide, we developed IPGRM, a novel approach leveraging indel pattern regularity for efficient repair gene discovery (Fig. 3A–C). IPGRM was implemented by first constructing a genome-wide knockout library in HEK293T cells using the human GeCKOv2 library, followed by secondary editing at two target sites (sg3 and sg81) adjacent to library sgRNAs. Amplicon sequencing of these edited sites enabled simultaneous capture of repair outcomes along with library sgRNA identities, enabling high-throughput mapping of genetic perturbations to specific repair phenotypes. Quality control of the IPGRM libraries indicated robust screening suitability (Supplementary Fig. S6). sgRNA sequencing achieved ∼90% coverage, with read counts predominantly distributed between 100 and 300 (Supplementary Fig. S6C–H). Moreover, sgRNA abundances were highly correlated between sg3 and sg81 libraries, confirming library consistency (Supplementary Fig. S6I). These metrics collectively validate the technical quality of IPGRM for genome-wide repair analysis.

Analysis of indel patterns in the IPGRM libraries recapitulated the characteristic repair signatures observed in our initial studies (Fig. 3J and Supplementary Fig. S7). Insertions were predominantly 1 bp, with a substantial fraction templated from flanking nucleotides (Fig. 3D and E and Supplementary Fig. S7B and C). Deletions represented the major outcome (83.3% for sg3 and 66.6% for sg81, Supplementary Fig. S7A), with small deletions (≤5 bp) being most frequent (Fig. 3F and G). Microhomology analysis revealed that recurrent deletions (3 bp in sg3; 5 bp in sg81) precisely aligned with upstream MH sequences (Fig. 3H and I), implicating MMEJ as a key mechanism. Deletion breakpoints were enriched PAM-distally (Supplementary Fig. S7D and E), possibly reflecting persistent Cas9 binding [19]. Mutations were infrequent (5.5%–6.9%) and localized within 5 bp of the cleavage site, consistent with previous observations (Supplementary Fig. S7F and G). Together, these results validate IPGRM’s ability to capture biologically relevant repair patterns while enabling genome-scale screening.

Our sgRNA screen revealed distinct gene signatures associated with each of the seven indel patterns (Supplementary Figs S8 and S9). One-base-pair insertions were significantly reduced upon knockout of core NHEJ factors (e.g. LIG4, PRKDC, XRCC5/6, POLL, NHEJ1), underscoring their essential role in promoting rapid end-joining (Supplementary Figs S8A and S9A). In contrast, depletion of HR-associated genes (e.g. MRE11A, NBN, RBBP8) increased 1 bp Ins frequency (Supplementary Figs S8A and S9A), suggesting that early HR initiation exerts a competitive suppressive effect on NHEJ. A parallel trend was observed for Large Ins, where NHEJ factors, ranging from DNA end processing to ligation, acted as positive regulators, whereas end-resection factors in HR (e.g. RBBP8, RAD50) suppressed this outcome (Supplementary Figs S8B and S9B). At the individual sgRNA level, we observed that genes associated with end resection (e.g. MRE11A, NBN) and the canonical MMEJ pathway (e.g. POLQ, LIG3, PARP1) showed modulating trends on the MH Small/Large Del classes at specific target sites (e.g. sg3, sg81) (Supplementary Figs S8C and D and S9C and D), consistent with the known mechanism of this repair type. However, in the integrated gene-level significance analysis encompassing all sgRNAs, these canonical factors did not emerge as top significant hits (Supplementary Table S2). In contrast, NonMH Small Del were positively regulated by classical NHEJ components (e.g. PRKDC, XRCC6, POLM), whereas HR gene knockouts further increased their prevalence, consistent with pathway competition (Supplementary Figs S8E and S9E). Strikingly, NonMH Large Del displayed inverted regulation: DNA end-protection factors in NHEJ (e.g. PRKDC, RIF1) suppressed this outcome, whereas HR-associated resection initiators (e.g. MRE11A, RAD50) promoted it, implying a resection-dependent mechanism distinct from MMEJ (Supplementary Figs S8F and S9F). Finally, Mut were linked to replication and mismatch repair genes (e.g. RPA1, EXO1, MSH6), suggesting involvement of atypical repair routes (Supplementary Figs S8G and S9G).

**Figure 4.**
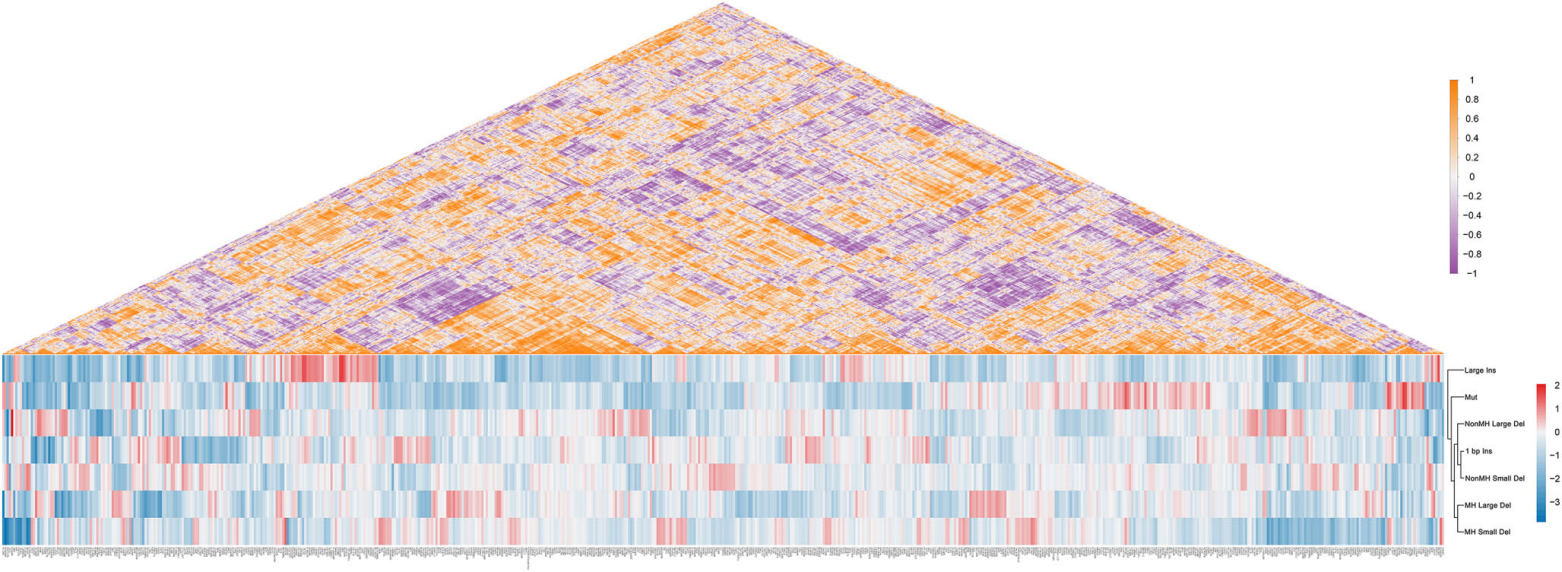
IPGRM enables data-driven inference of the genetic organization of DSB indel patterns. The lower heatmap shows hierarchical clustering of standardized log₂ FC values for seven repair patterns across the top 50 genes identified as repair modulators. Functional groupings of repair outcome patterns are indicated by the dendrogram on the right, constructed using the average-linkage clustering method. The upper triangular heatmap displays pairwise Pearson correlation coefficients of gene-level repair outcome signatures from the IPGRM top 50 DEGs dataset. Strong positive and negative correlations reveal cooperative and antagonistic repair modules.

Hierarchical clustering of repair outcomes based on gene-regulatory profiles revealed clear mechanistic partitioning (Fig. 4). MH-associated deletions formed a tight cluster, reflecting their shared dependence on MMEJ. One-base-pair insertions, NonMH Small Del, and NonMH Large Del were positioned in a partially overlapping branch, consistent with their reliance on NHEJ factors. In contrast, Large Ins and Mut constituted distinct branches, suggesting involvement of distinct mechanisms. To further characterize these two atypical branches, we examined the associated gene signatures. The Large Ins branch showed a polymerase-associated profile, with polymerase-related regulators, including PRIMPOL and POLD4 among the significant hits, together with additional polymerases such as POLK and POLM (Supplementary Fig. S10A). In contrast, the Mut branch was enriched for chromatin regulators and remodeler-related factors, including the canonical regulators KMT2A, HDAC2, and CBX5, as well as chromatin-associated factors such as L3MBTL1 and ZMYND8, together with the histone genes H2AC8 and H2BC14. In addition, replication- and cell cycle–associated genes, including MCM7, CCNA1, and CENPE, as well as DNA repair factors such as RECQL5 and ERCC4/ERCC6, were also identified (Supplementary Fig. S10C).

Pearson correlation coefficients were calculated between every pair of genes to evaluate the similarity of their repair outcome profiles. Examination of the correlation matrix revealed clusters of genes with highly similar regulatory patterns, suggestive of potential cooperative modules, as well as pairs of genes with opposing patterns, indicative of possible antagonistic relationships. These results highlight that repair-associated genes are not acting independently but instead form correlation-based networks, providing a global view of cooperative and competitive interactions underlying diverse repair outcomes. Together, these analyses establish a genome-wide repair atlas that mechanistically validates our seven-class outcome framework and provides a foundation for predicting and manipulating repair outcomes in genome editing applications.

### IPGRM Identifies miRNA and protein-coding networks governing distinct indel patterns in CRISPR/Cas9 editing

**Figure 5.**
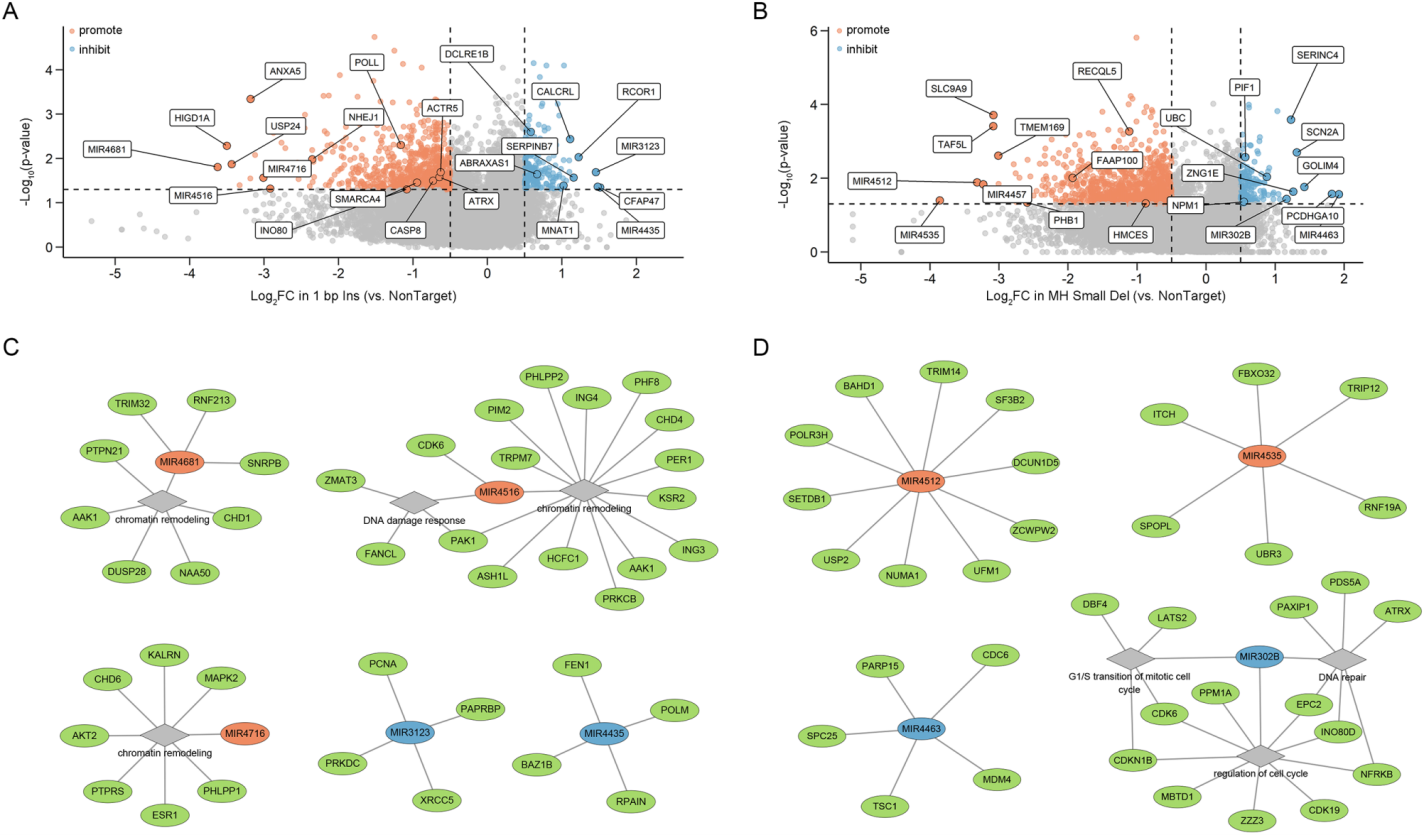
IPGRM uncovers multi-layered regulation of DSB repair by protein-coding genes and miRNAs. Volcano plots showing genome-wide screening results associated with 1 bp Ins (**A**) and MH Small Del (**B**) repair patterns. Each point represents a gene or miRNA, with *x*-axis indicating log₂ fold change (versus NonTarget controls) and *y*-axis showing –log₁₀ *P*-values. Orange and blue indicate factors promoting or inhibiting the respective repair types. Representative regulators are labeled. Dashed lines mark significance thresholds. Predicted miRNA–target interaction networks for representative miRNAs associated with 1 bp Ins (**C**) or MH Small Del (**D**). miRNAs and their predicted targets were identified using TargetScan and miRDB.

Using IPGRM, we systematically identified genes specifically associated with distinct indel patterns, including miRNA genes, protein-coding genes, and direct repair effectors (Fig. 5 and Supplementary Fig. S10). Notably, both miRNA-encoding loci and classical repair genes exhibited outcome-specific enrichment patterns, suggesting layered regulatory networks. For instance, miRNAs predominantly influenced repair bias through transcript-level modulation of repair factors, while protein-coding genes were enriched in functions related to genome stability and cellular regulation. These findings suggest that repair outcome determination is governed by the interplay between transcriptional regulation and direct repair activities.

Strikingly, top-ranked genes across repair outcomes included numerous miRNAs, which were associated with shifts in repair frequencies through their predicted targeting of repair-related mRNAs (Fig. 5A–D). In 1 bp insertion–prone edits, promoting miRNAs such as MIR4681, MIR4516, and MIR4716 were significantly enriched, with predicted targets broadly involved in chromatin remodeling and mRNA splicing regulation; for example, MIR4681 is predicted to target SNRPB, a splicing regulator of POLA1 and BRCA2, thereby suppressing HR and favoring 1-bp insertions [20]. Conversely, miRNAs enriched in 1 bp insertion–inhibiting genes, including MIR3123 and MIR4435, were predicted to target key NHEJ factors such as POLM, PRKDC, and XRCC5 (Fig. 5A and C). Notably, the most significant hits for the MH deletion module were enriched for putative transcriptional regulators and multiple miRNAs, and the predicted targets of these miRNAs were themselves enriched in DNA repair and cell cycle pathways, suggesting a layer of post-transcriptional control over MMEJ-associated outcomes. For MH Small Del, promoting miRNAs included MIR4512 and MIR4535, with predicted targets involved in end-joining and resection processes, whereas inhibiting miRNAs, such as MIR302B and MIR4463, were enriched for cell cycle regulators, including CDC6, whose suppression can reduce S-phase entry and potentially limit MMEJ (Fig. 5B and D) [21]. Together, these findings underscore miRNAs as pivotal players in fine-tuning repair pathway choice via multi-gene regulatory cascades.

Beyond miRNAs, protein-coding genes involved in DNA metabolism were robustly enriched (Fig. 5A and B and Supplementary Fig. S10). Functional annotation highlighted roles in end resection (e.g. EXO1, EXD2), helicase-mediated repair (e.g. RECQL5, PIF1), specialized polymerases (e.g. POLQ, POLM, POLK), core NHEJ components (e.g. NHEJ1, DCLRE1C, APLF), and chromatin remodeling (e.g. SMARCAD1, NPM1). In addition, our hits also included factors that may act upstream as regulators of canonical repair machinery. For example, PHB1 was identified among the MH Del–promoting hits (Fig. 5B), and prior studies have reported that PHB1 depletion reduces FOXM1 levels [22]. FOXM1 has been described as a cofactor of LIG3 and a transcriptional regulator of XRCC1, and it can stimulate the expression of multiple DNA repair factors [23]. PPI analysis of 1 bp Ins–promoting and MH Small Del–inhibiting genes revealed several functional modules (Supplementary Fig. S11). Survival analysis of these modules showed that genes promoting 1 bp Ins (e.g. CASP8, SMARCA4, INO80) significantly correlated with poor breast cancer patient survival (HR = 1.3–2.0), implicating rapid error-prone repair in tumor adaptation (Supplementary Fig. S12A–E). Conversely, MH Small Del suppressors (e.g. S100A8, MTREX) are associated with improved outcomes (HR = 0.6–0.8), suggesting MMEJ inhibition as a potential therapeutic strategy (Supplementary Fig. S12F–J). The convergence of direct repair effectors and indirect regulators provides a foundation for mechanistic studies and therapeutic targeting of repair networks in genome editing and disease contexts.

### S100A8 decreases microhomology-mediated small deletion by inhibiting MMEJ pathway

**Figure 6.**
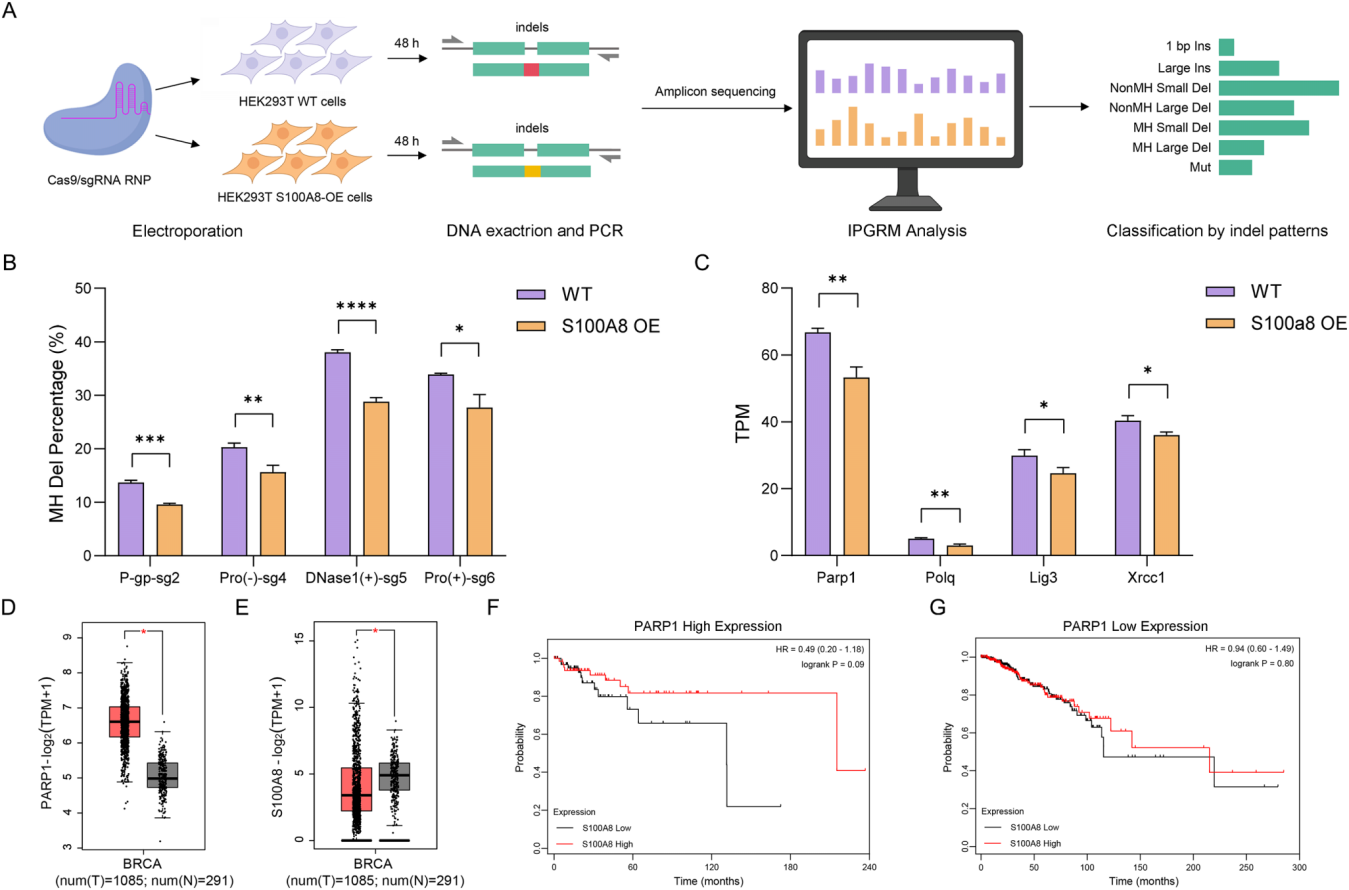
S100A8 overexpression suppresses MH deletions and modulates MMEJ-associated factors. (**A**) Schematic overview of the validation workflow. HEK293T wild-type (WT) and S100A8-overexpressing (S100A8-OE) cells were electroporated with Cas9/sgRNA RNP complexes targeting indicated loci, followed by DNA extraction, amplicon sequencing, and IPGRM analysis. (**B**) Quantification of MH-mediated deletion percentages across four representative target sites in WT and S100A8-OE cells. *n* = 3, *P ≤ .05, ***P  <  .001, ****P < .0001. (**C**) Expression levels of MMEJ-related genes (Parp1, Polq, Lig3, and Xrcc1) in WT and S100a8-OE cells measured by RNA-seq. (**D, E**) GEPIA2 breast cancer (BRCA) dataset showing differential expression of PARP1 and S100A8 between tumor (T, *n* = 1085) and normal (N, *n* = 291) samples. (**F, G**) Kaplan–Meier survival analysis of breast cancer patients stratified by high and low S100A8 expression in PARP1 high and low subgroups, respectively. Statistical significance was assessed by log-rank test.

Our IPGRM analysis identified S100A8 as a potential regulator of DNA repair, although prior studies have reported conflicting roles in different cancer contexts. For example, S100A8 has been described as a chemosensitization target in esophageal squamous cell carcinoma [24], yet it has been shown to suppress proliferation by activating the G2/M DNA damage response in head and neck cancer [25]. To clarify its role, we generated single-cell clones overexpressing S100A8 (Supplementary Fig. S13) and performed gene editing at four loci across distinct chromatin environments (Fig. 6A). IPGRM-based classification revealed that S100A8 overexpression consistently reduced MH Dels at all tested sites, while its effects on other indel patterns varied by target site (Fig. 6B and Supplementary Fig. S14). These findings strongly support S100A8’s specific inhibitory role in MMEJ pathway.

To elucidate the mechanism of S100A8-mediated MMEJ suppression, we analyzed GEO datasets from S100a8-overexpressing mouse cells. S100a8 overexpression significantly downregulated core MMEJ factors, including PARP1, POLQ, LIG3, and XRCC1 (Fig. 6C). Subsequent analysis of GEPIA2 data further revealed an inverse correlation between S100A8 and PARP1 expression in breast cancer versus normal tissues (Fig. 6D and E and Supplementary Fig. S15). Importantly, TCGA-based survival analysis showed that high S100A8 expression correlated with better prognosis specifically in PARP1-high breast cancer patients (HR = 0.49, *P *= .09), but not in PARP1-low cases (HR = 0.94, *P *= .80) (Fig. 6F and G). These results suggest a functional interaction between S100A8 and PARP1 in regulating MMEJ activity and influencing chemotherapy response.

Integrating our experimental findings with existing literature, we propose a mechanistic model of DSB repair pathway regulation. This model highlights how IPGRM-identified genes such as S100A8 differentially modulate repair outcomes, with S100A8 specifically suppressing MMEJ through downregulation of key factors including PARP1 (Supplementary Fig. S16). Our results help reconcile previous contradictory reports on S100A8’s role in DNA repair and provide new insights into how its expression status may serve as a predictive biomarker for PARP inhibitor sensitivity in breast cancer. The comprehensive characterization of S100A8’s function underscores IPGRM’s power in bridging mechanistic discoveries with potential clinical applications.

## Discussion

In this study, we present IPGRM, an innovative computational-experimental framework for systematically deciphering the complex regulatory networks that govern DSB repair following CRISPR/Cas9 editing. IPGRM integrates three critical dimensions of repair analysis: (i) comprehensive outcome spectra, (ii) kinetic dynamics across timepoints, and (iii) functional gene regulatory networks, thereby establishing a multidimensional atlas of DSB repair mechanisms. Through large-scale pattern analysis, we identified seven distinct indel subtypes that represent fundamental repair modalities. Each subtype exhibits characteristic sequence features and temporal behaviors, with NHEJ-associated 1 bp insertions arising early and MMEJ-associated deletions emerging more gradually. Network-level analysis systematically identified genes specifically associated with distinct CRISPR/Cas9-induced repair outcomes, including miRNA genes, protein-coding genes, and direct repair effectors. Together, these findings provide a robust framework for mechanistic studies of genome editing and open new avenues for precision control of repair outcomes in therapeutic applications.

Our study first revealed that Cas9-mediated editing outcomes follow striking regularity, with distinct kinetic signatures across different indel patterns. Predictable outcomes such as templated 1 bp Ins and MH Dels constitute a substantial fraction of repair events and underscore the conserved and non-random nature of DSB resolution. The prevalence of templated 1 bp Ins may stem from the single-base 5′ overhang generated by Cas9 cleavage, which can transiently serve as a template for fill-in synthesis, as suggested by prior studies of Cas9 end processing [10–12, 26]. Likewise, the overwhelming bias toward MH Dels highlights the genome’s reliance on local microhomologies to guide break resolution, consistent with reports that MMEJ-dominated repair is favored in chromatin contexts with accessible microhomologous flanks [10, 27]. Kinetic analyses further demonstrate how these mechanistic differences translate into temporal patterns of repair. MH Dels accumulate more slowly (24–48 h), with longer microhomologies further delaying completion, whereas NonMH Dels resolve hierarchically, with small indels (<5 bp) closing within 3 h and larger deletions (>5 bp) persisting for 12–18 h. Together with our network analysis implicating histone modifiers and DNA helicases as regulators of repair choice, these results support a model in which both DNA end configuration and chromatin environment jointly determine repair pathway selection and kinetics [28, 29].

Beyond structural determinants, our study highlights that miRNAs play critical roles in regulating DNA repair pathway choices. Through IPGRM screening, we uncovered a significant enrichment of specific miRNAs in distinct repair outcomes, revealing their ability to bias repair by simultaneously targeting multiple repair-related genes. Unlike conventional CRISPR screens that focus primarily on protein-coding genes, our data demonstrate that miRNAs form an additional regulatory layer with remarkable specificity. Many of these miRNAs have previously been linked to cancer progression and therapy resistance [30–32], consistent with reports that tumor-associated miRNAs often promote genomic instability by disrupting DNA repair [33, 34]. For instance, MIR4516, which promotes 1 bp Ins, is predicted to suppress FANCL, a key HR repair factor known to facilitate end resection and homologous recombination [35], thereby biasing repair toward NHEJ insertion. Conversely, MIR302B, which antagonizes MMEJ, is predicted to target CDK6, a cyclin-dependent kinase frequently upregulated in cancer and implicated in therapy resistance. Suppression of CDK6 has been reported to impair HR, while its impact on NHEJ remains context-dependent [36], thereby shifting the balance of repair pathway choice. More broadly, our screening data suggest that indel patterns are influenced by a complex regulatory network. This network includes known core repair components, which show functional associations at the single-sgRNA level, but is prominently populated by a series of novel regulators. The discovery of these novel factors reveals a previously underappreciated layer of molecular regulation that fine-tunes repair pathways such as MMEJ. These findings position miRNAs as key regulators of repair pathway choice by fine-tuning critical effector genes, creating regulatory nodes that influence genomic stability. While previous studies have linked individual miRNAs to DNA repair, our genome-wide data provides a comprehensive view of how miRNA networks coordinately shape repair outcomes. The cancer-relevant functions of these miRNAs further suggest their potential utility as biomarkers or therapeutic targets, particularly for tumors dependent on specific repair vulnerabilities. This work significantly expands our understanding of the complex regulation of DNA repair and opens new avenues for miRNA-based strategies in genome editing and cancer therapy.

Similarly unexpected was our discovery that S100A8, traditionally characterized as an inflammatory mediator, also plays a direct role in DNA repair pathway regulation. Previous studies have established that S100A8, primarily secreted by immune cells as a heterodimer with S100A9, acts as an inflammatory mediator. It promotes tumor cell proliferation and inhibits apoptosis in the tumor microenvironment [37], and also exhibits antimicrobial activity against pathogens such as *Staphylococcus aureus* [38]. In contrast to these extracellular functions, we demonstrate for the first time that S100A8 significantly suppresses MMEJ by downregulating PARP1 expression. This newly identified role contrasts with prior studies that emphasized only its extracellular activities and aligns with emerging evidence that inflammatory mediators can influence DNA repair processes [39]. Importantly, survival analysis in HR-deficient breast cancer patients suggests that S100A8-mediated PARP1 suppression, and consequent MMEJ inhibition, may contribute to chemotherapy response and survival outcomes, potentially explaining associations between high S100A8 levels and better prognosis in specific cancer subtypes [25]. These findings bridge the gap between inflammation-associated proteins and DNA repair regulation, proposing a mechanism by which S100A8 might promote genomic instability in chronic inflammatory conditions while simultaneously creating a therapeutic vulnerability in HR-deficient cancers. Future studies should explore whether targeting the S100A8–PARP1 axis could sensitize tumors to DNA-damaging agents, particularly in inflammatory microenvironments where S100A8 is highly expressed.

Our indel pattern classification was developed for Cas9-induced DSB repair, but it is defined by general outcome features and should be portable beyond the loci and cell line studied here. Applying it to other DSB editors such as Cas12a will require re-estimating pattern boundaries and kinetic parameters because differences in end structures can shift indel architectures, and across cell types or species the same pattern definitions may hold even if their frequencies and kinetics change with repair activity and cell-cycle state. Extending IPGRM to base editors or prime editors will require more substantive adaptation because their outcome is dominated by substitutions and editor-specific byproducts, necessitating redefinition of outcome classes and re-parameterization of the kinetic model. Finally, our genome-wide screens were performed in 293T cells to leverage high transfection efficiency and robust editing frequencies. However, 293T cells have well-recognized limitations for DNA damage response studies: SV40 large T antigen alters replication and cell-cycle control [40], and reported mismatch-repair defects (including MLH1 deficiency) [41], together with cell-line–specific alterations in DDR components (e.g. MRN-related differences) [42], may influence absolute outcome distributions. While these features are unlikely to alter the core regulatory relationships identified here, they may affect quantitative outcome frequencies. Therefore, systematic validation in additional cellular backgrounds, including non-transformed and repair-proficient cells, will be important to assess the generality of these findings.

During the submission of this work, a genome-wide Repair-seq study (REPAIRome) was published, underscoring the growing emphasis on systematic, large-scale approaches for dissecting DSB repair networks [43]. In this context, we compared our global repair profiles with the dataset from that study to gauge cross-study concordance. Overall, the major outcome signatures appeared broadly consistent with those observed in IPGRM, including dominant 1-bp insertions and enriched microhomology-associated deletions (Supplementary Fig. S17A–D). We also applied the analysis workflow described in REPAIRome to our IPGRM dataset; for instance, POLL knockout increased microhomology-associated deletions, producing an interpretable outcome shift (Supplementary Fig. S17E–H). Taken together, these comparative analyses support outcome reproducibility and reveal unique gene-class associations specific to our framework, demonstrating the complementary and distinctive value of IPGRM in mapping DNA repair regulatory networks.

Collectively, these findings establish CRISPR repair outcomes as a quantifiable phenotype emerging from multilayered regulation. Our IPGRM framework demonstrates that sequence features and kinetic parameters interact with higher-order regulatory layers, including miRNA networks and inflammatory signals, to jointly determine editing outcomes. This advances the field beyond chromatin-centric models of repair control. Future work should explore (i) single-molecule dynamics of identified regulators, (ii) physiological contexts of inflammation–repair crosstalk, and (iii) strategies for therapeutic targeting of these pathways. This systems-level understanding enables both precision genome engineering and innovative cancer therapies that exploit repair network vulnerabilities.

## Supplementary Material

gkag260_Supplemental_Files

## Data Availability

The sequencing data for the sg3 and sg81 libraries generated in this study have been deposited in the NCBI BioProject database under accession number PRJNA1348960. Process files and custom Python scripts used for data processing and analysis are available on Figshare (DOI: 10.6084/m9.figshare.30451736). Other data supporting the findings of this study are available from the corresponding author upon reasonable request.
