# Supplementary material for: Indel pattern-guided repair mapping reveals genome-wide DNA repair networks in CRISPR/Cas9 editing
Source: Nucleic Acids Res. 2026 Mar 27;54(6):gkag260. doi: 10.1093/nar/gkag260 (PMC13023040; doi:10.1093/nar/gkag260)
Supplement: gkag260_Supplemental_Files [file gkag260_supplemental_files.zip › Supplementary Figures.docx]

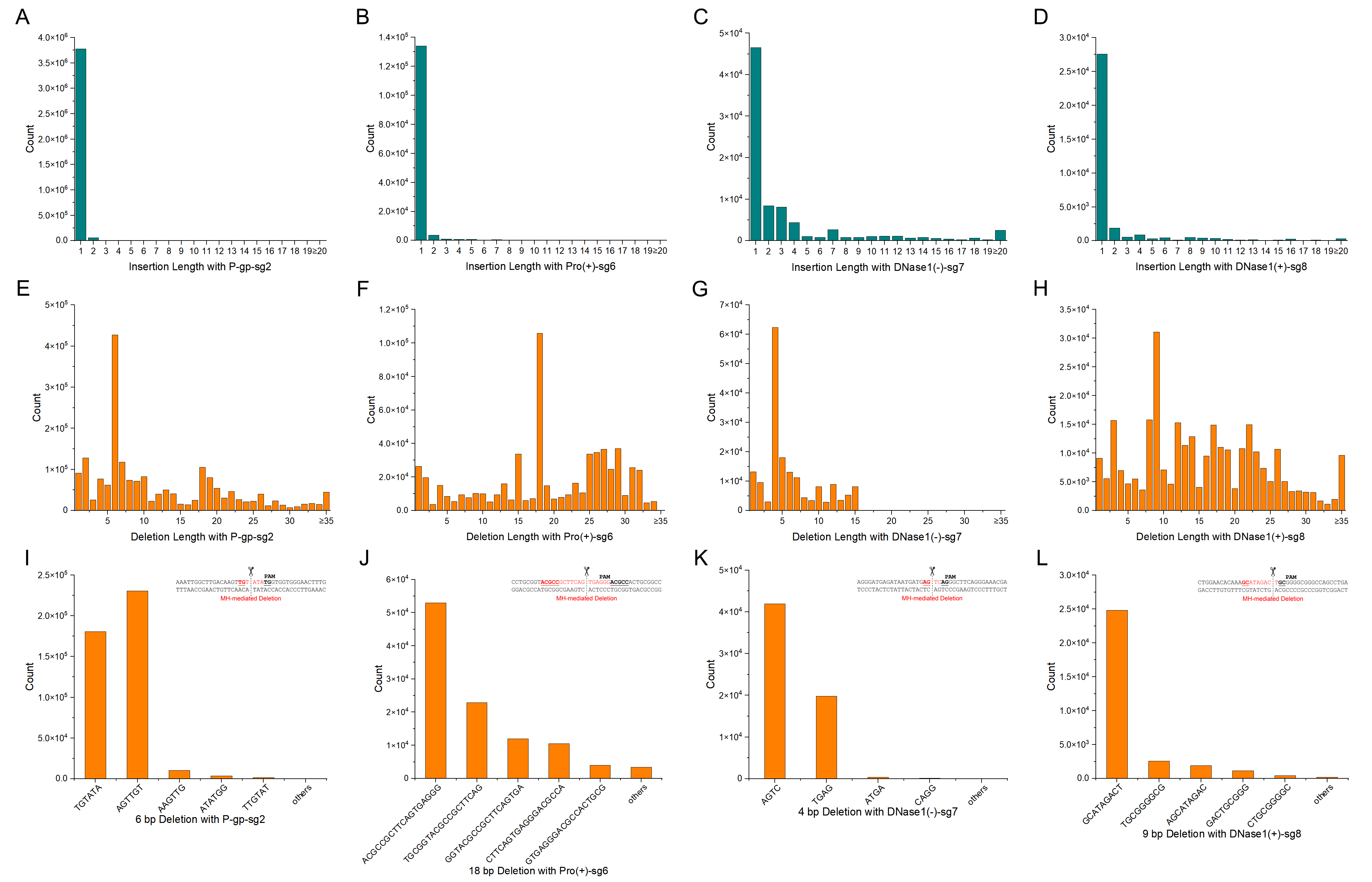


**Supplementary Figure S1.** Distribution of Cas9-induced indel outcomes at additional target loci. (**A–D**) Insertion length distribution at P-gp-sg2 (**A**), Pro(+)-sg6 (**B**), DNaseI(+)-sg7 (**C**), and DNaseI(+)-sg8 (**D)**. (**E–H**) Deletion length distribution at P-gp-sg2 (**E**), Pro(+)-sg6 (**F**), DNaseI(+)-sg7 (**G**), and DNaseI(+)-sg8 (**H**). (**I–L**) Sequence profiles of the most frequent deletions: 6 bp at P-gp-sg2 (**I**), 18 bp at Pro(+)-sg6 (**J**), 4 bp at DNaseI(-)-sg7 (**K**), and 9 bp at DNaseI(+)-sg8 (**L**). Microhomologous bases are indicated by underlining.


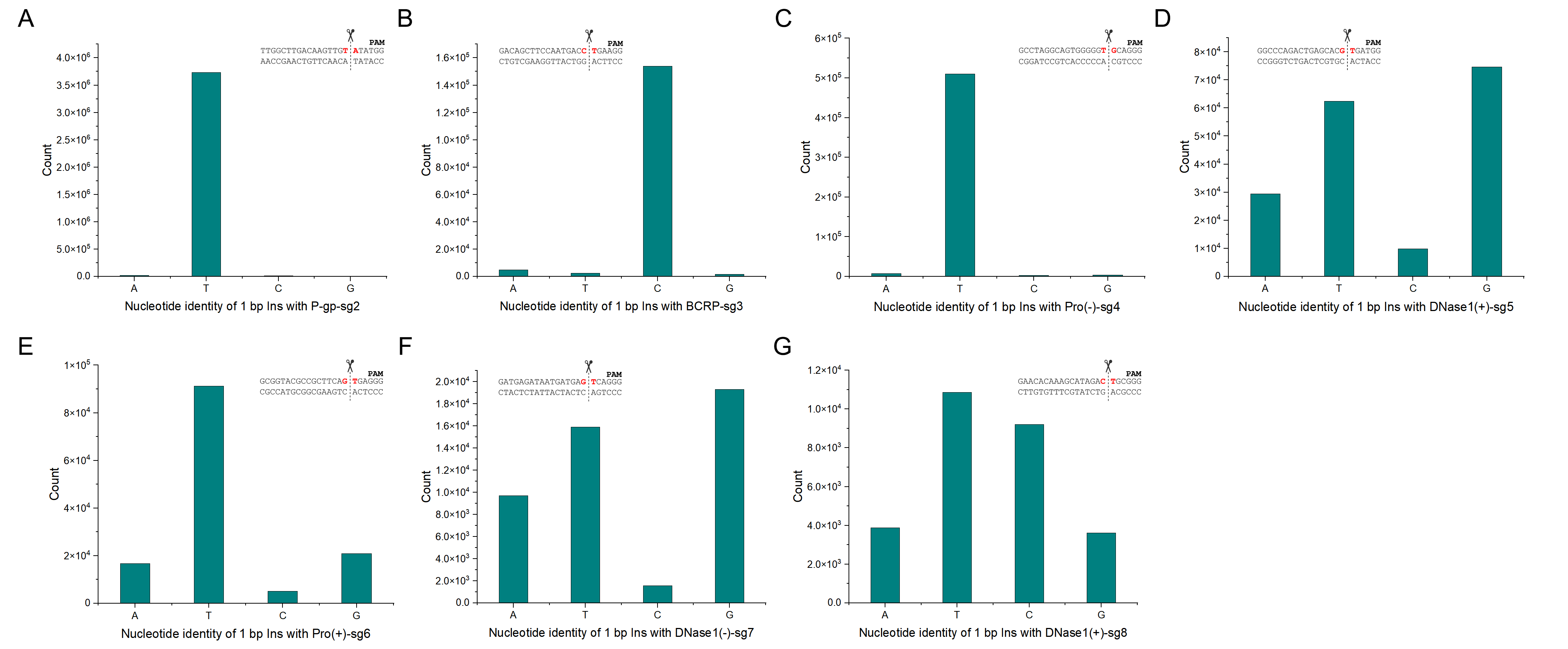


**Supplementary Figure S2.** Nucleotide identity of 1 bp insertions across seven target loci. (**A–G**) Distribution of inserted nucleotides (A, T, C, G) among 1 bp insertion events at P-gp-sg2 (**A**), BCRP-sg3 (**B**), Pro(-)-sg4 (**C**), DNaseI(+)-sg5 (**D**), Pro(+)-sg6 (**E**), DNaseI(-)-sg7 (**F**), and DNaseI(+)-sg8 (**G**). The nucleotides flanking the cleavage site are highlighted in red.


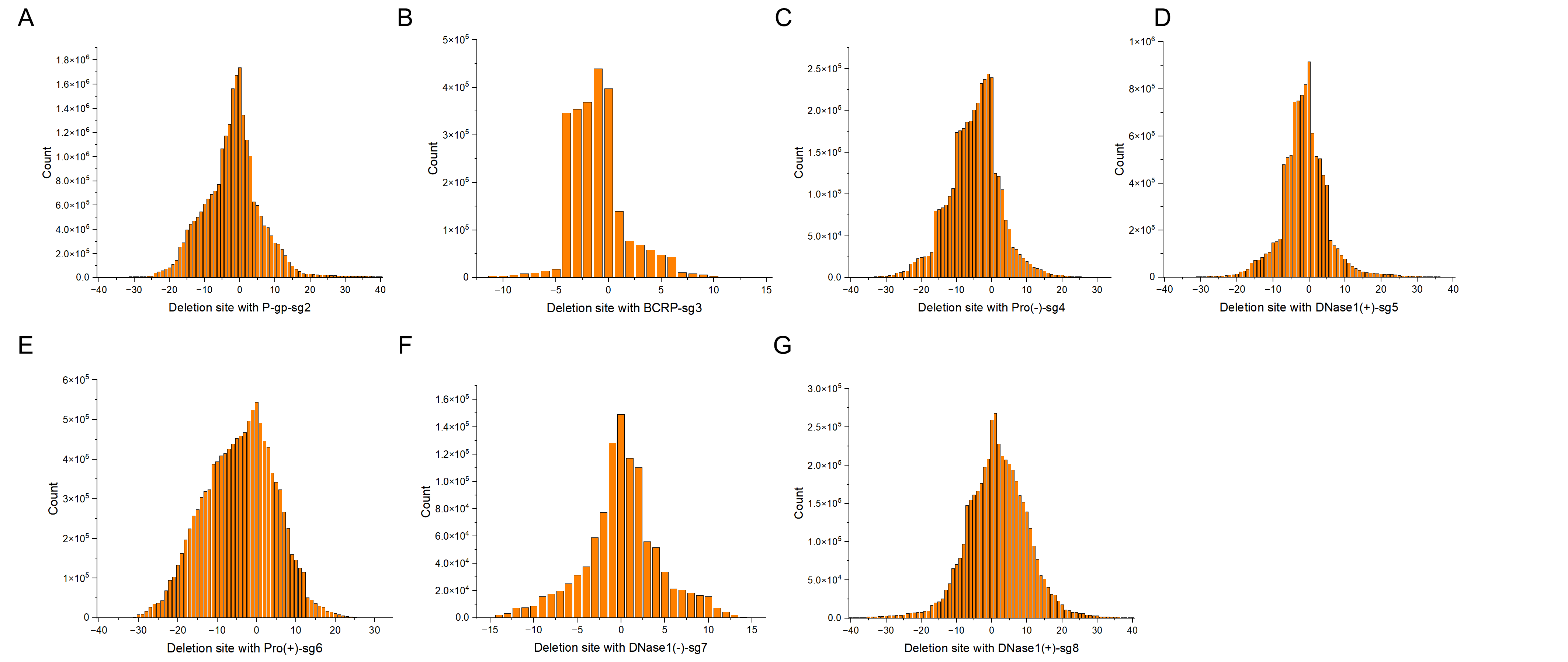


**Supplementary Figure S3.** Distribution of deletion sites across seven target loci. (**A**) P-gp-sg2, (**B**) BCRP-sg3, (**C**) Pro(–)-sg4, (**D**) DNaseI(+)-sg5, (**E**) Pro(+)-sg6, (**F**) DNaseI(–)-sg7, (**G**) DNaseI(+)-sg8. **Deletion site** indicates nucleotide positions covered by deletion events, where each covered base is counted once. The fourth base upstream of the PAM is defined as position 0.


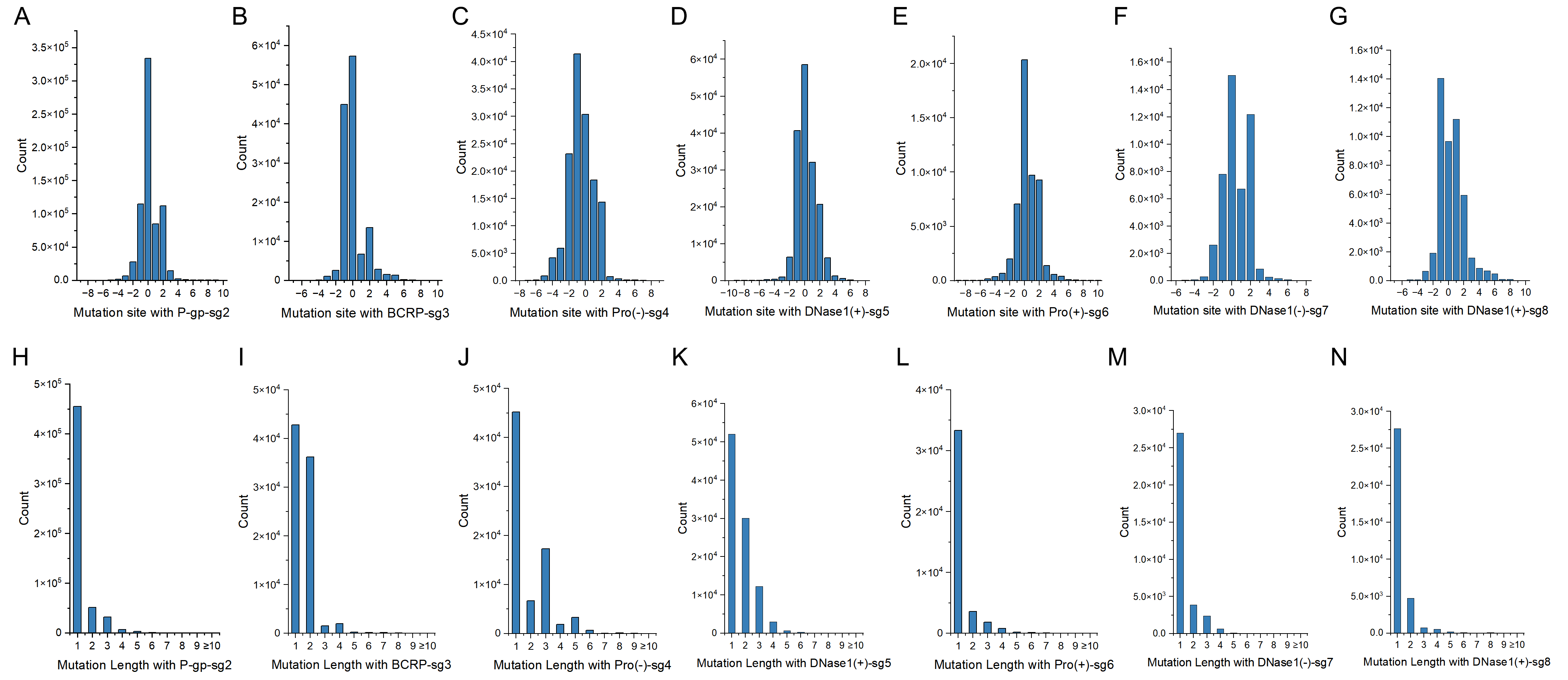


**Supplementary Figure S4.** Analysis of mutation repair outcomes across seven target loci. (**A–G**) Distribution of mutation sites at P-gp-sg2 (**A**), BCRP-sg3 (**B**), Pro(–)-sg4 (**C**), DNaseI(+)-sg5 (**D**), Pro(+)-sg6 (**E)**, DNaseI(–)-sg7 (**F**), and DNaseI(+)-sg8 (**G**). (**H–N**) Distribution of mutation lengths at P-gp-sg2 (**H**), BCRP-sg3 (**I**), Pro(–)-sg4 (**J**), DNaseI(+)-sg5 (**K**), Pro(+)-sg6 (**L**), DNaseI(–)-sg7 (**M**), and DNaseI(+)-sg8 (**N**).


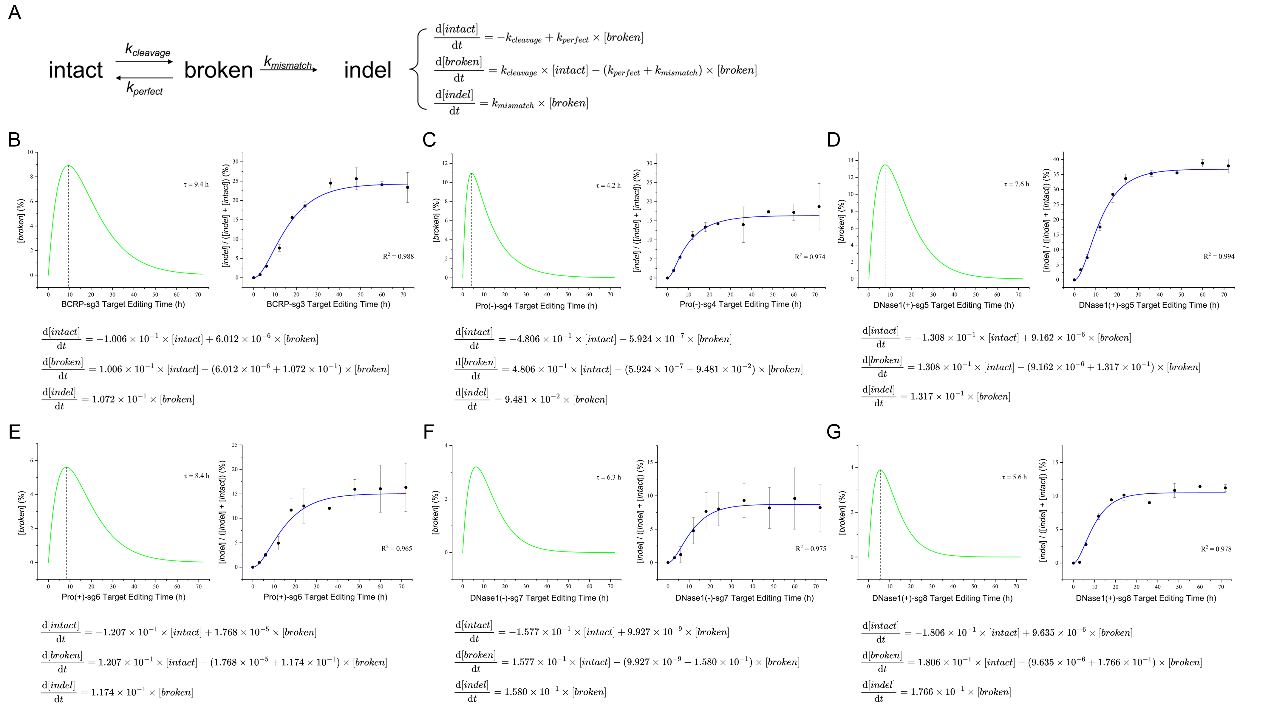


**Supplementary Figure S5.** Kinetic modeling of Cas9-induced DSB repair. (**A**) Schematic of the ordinary differential equation (ODE) framework used to model the dynamics of intact, broken, and indel alleles. Parameters include cleavage rate (k_cleavage_), error-free repair rate (k_perfect_), and error-prone repair rate (k_mismatch_). (**B–G**) Time-course fitting of editing outcomes at six genomic loci: BCRP-sg3 (**B**), Pro(–)-sg4 (**C**), DNaseI(+)-sg5 (**D**), Pro(+)-sg6 (**E**), DNaseI(–)-sg7 (**F**), and DNaseI(+)-sg8 (**G**). Left panels show accumulation of broken alleles; the τ denotes the timepoint of maximal broken allele accumulation. Right panels show observed indel accumulation (dots, experimental data) and fitted curves (lines, ODE model). Modeling was performed using an ODE-based framework adapted from Brinkman et al. (2018).


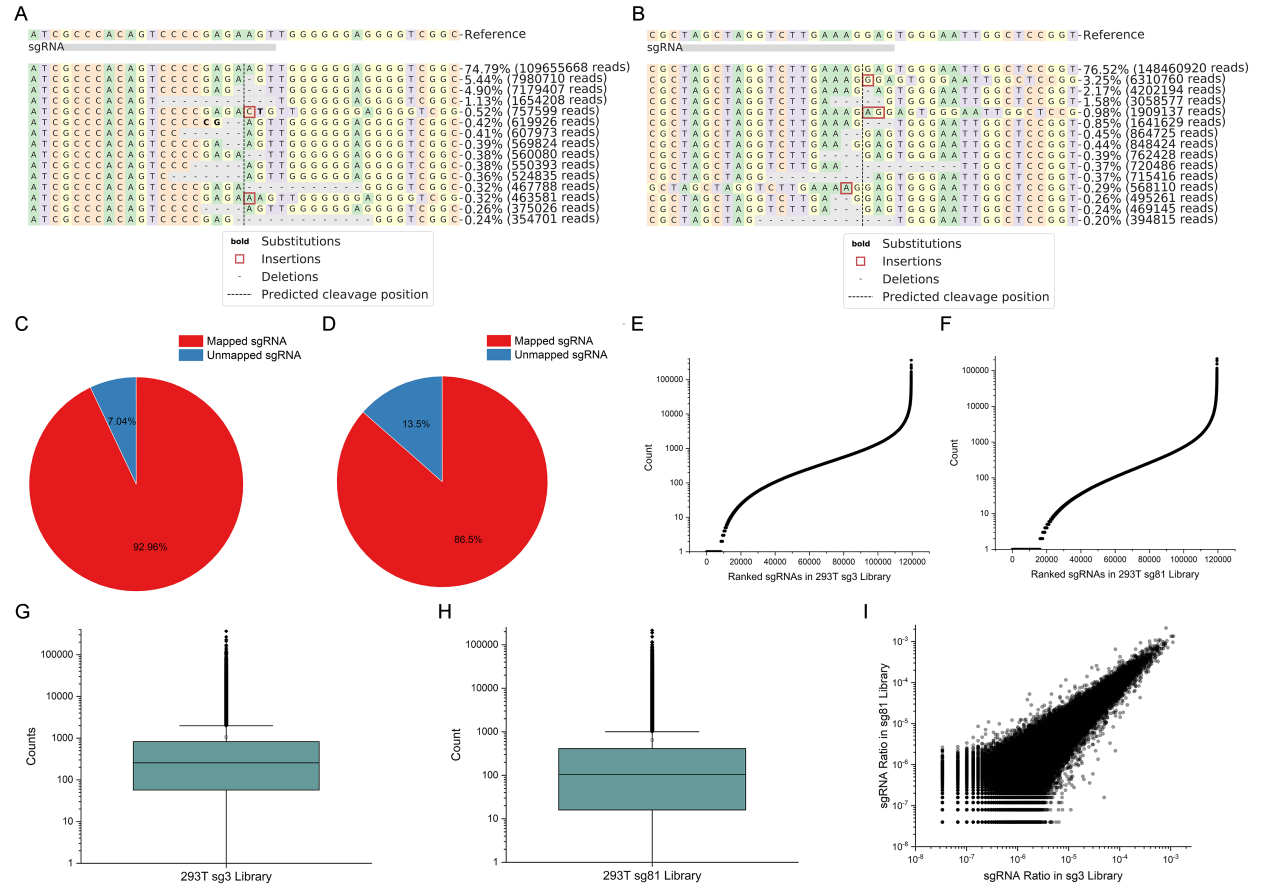


**Supplementary Figure S6.** Quality assessment of IPGRM sequencing libraries. (**A–B**) Representative CRISPResso2 analysis of editing outcomes at sg3 (**A**) and sg81 (**B**) loci, showing the most frequent indel and substitution types. Insertions, deletions, and substitutions are indicated, with the predicted Cas9 cleavage site marked. (**C–D**) Mapping efficiency of sequencing reads to library-encoded sgRNAs in the sg3 (**C**) and sg81 (**D**) libraries. (**E–F**) Distribution of sgRNA read counts across the sg3 (**E**) and sg81 (**F**) libraries. (**G–H**) Boxplots showing the distribution of sgRNA read counts in the sg3 (**G**) and sg81 (**H**) libraries. (**I**) Scatter plot showing the relative representation of sgRNAs in the sg3 versus sg81 libraries. Each point corresponds to a single sgRNA, with overall correlation between libraries indicated.


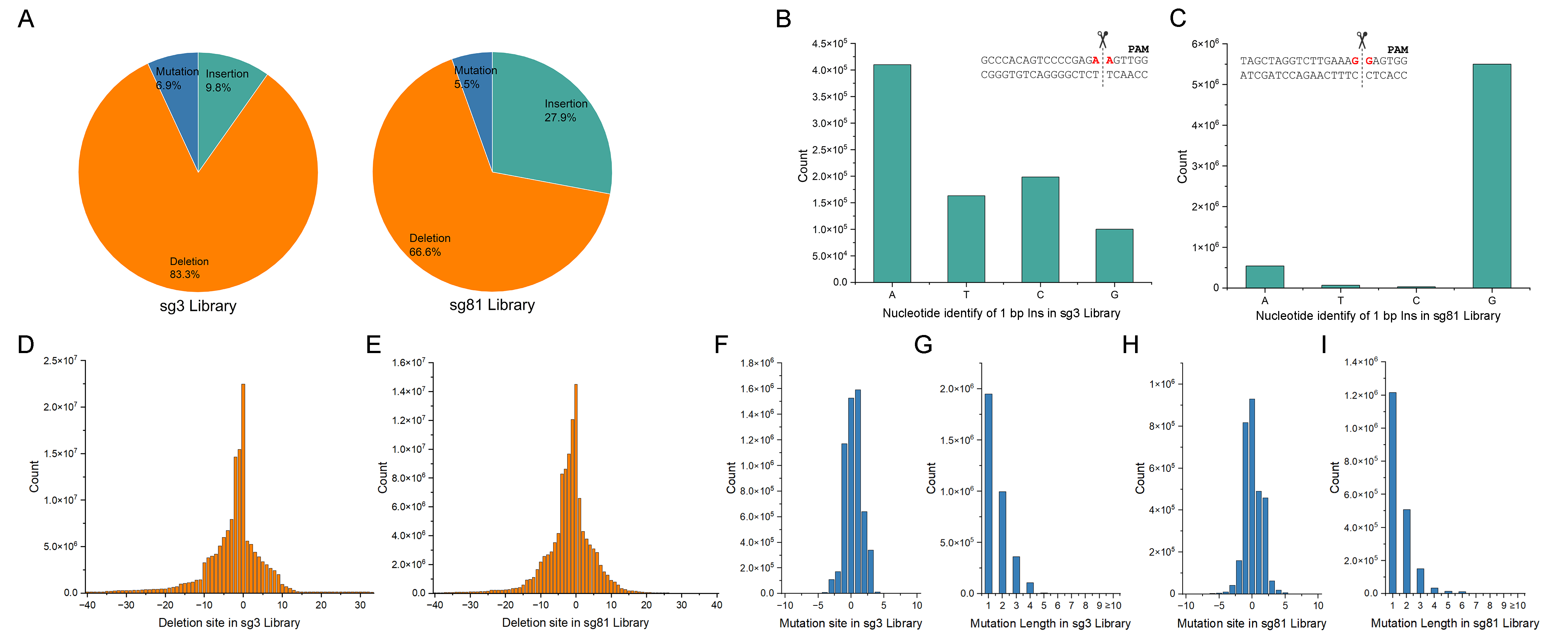


**Supplementary Figure S7.** Characterization of insertion, deletion, and mutation profiles in the sg3 and sg81 libraries. (**A**) Pie charts showing overall proportions of insertion, deletion, and substitution mutations in the sg3 and sg81 libraries. (**B–C**) Nucleotide identity distributions of 1 bp insertions in the sg3 library (**B**) and sg81 library (**C**). The cleavage site is indicated by scissors. (**D–E**) Distributions of deletion sites across the target region in the sg3 library (**D**) and sg81 library (**E**). The fourth base upstream of PAM is defined as position 0. (**F–G**) Mutation site distributions in the sg3 (**F**) and sg81 (**H**) libraries. (**G–I**) Mutation length distributions in the sg3 (**G**) and sg81 (**I**) libraries.


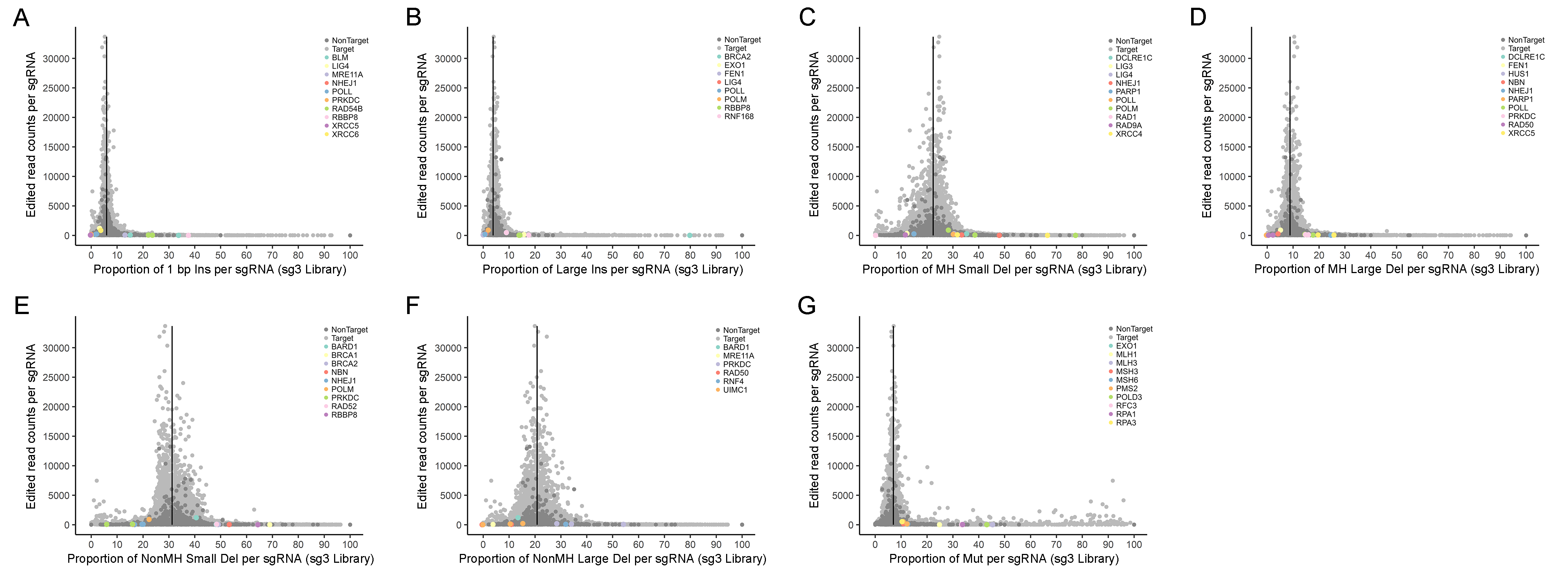


**Supplementary Figure S8.** sgRNA-level screening of repair outcome distributions in the IPGRM platform. Scatter plots show sgRNA-level distributions of repair patterns in the sg3 library. The x-axis indicates the proportion of a given repair outcome relative to all editing events for each sgRNA, and the y-axis indicates the total read count for that sgRNA. Each dot represents one sgRNA. A vertical black line denotes the mean proportion of non-targeting sgRNAs, serving as a reference baseline. (**A–G**) Distributions are shown for 1 bp Ins (**A**), Large Ins (**B**), MH Small Del (**C**), MH Large Del (**D**), NonMH Small Del (**E**), NonMH Large Del (**F**), and Mut (**G**). DSB repair regulators were highlighted and exhibited significant deviations from the overall distributions, confirming that IPGRM can sensitively capture pathway modulators at the sgRNA level.


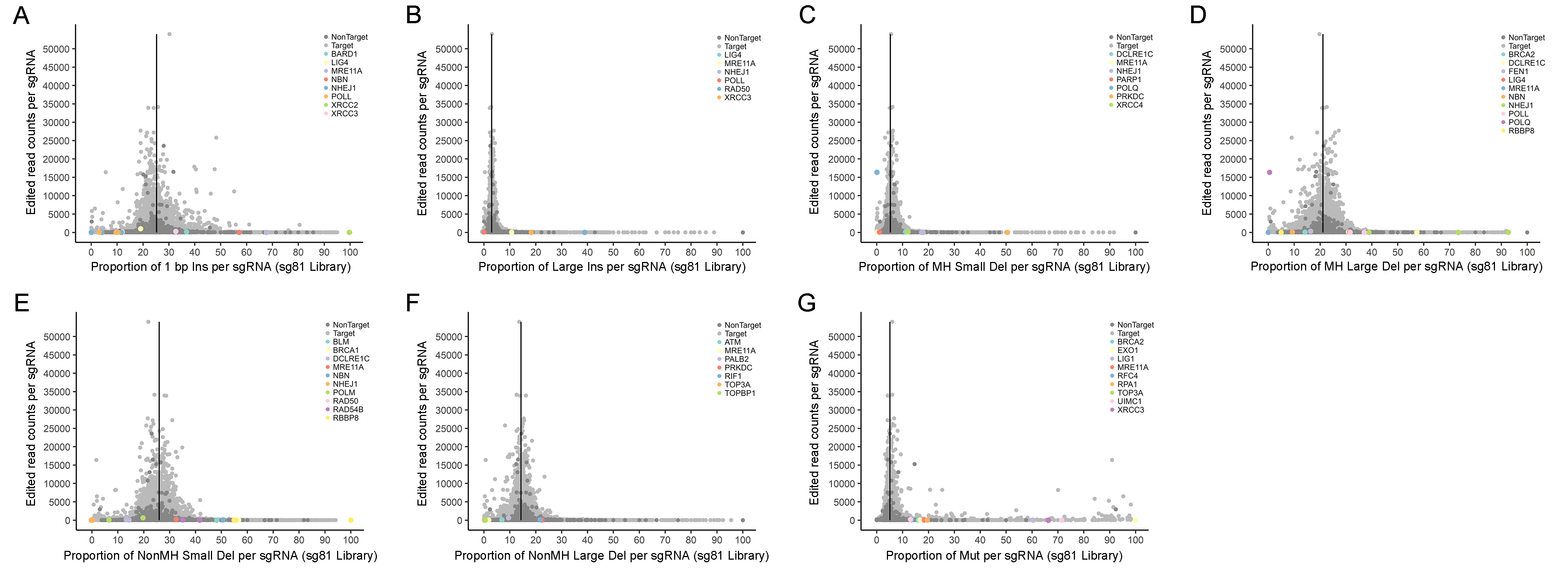


**Supplementary Figure S9.** sgRNA-level screening of repair outcome distributions in the IPGRM platform. Scatter plots show sgRNA-level distributions of repair patterns in the sg81 library. The x-axis indicates the proportion of a given repair outcome relative to all editing events for each sgRNA, and the y-axis indicates the total read count for that sgRNA. Each dot represents one sgRNA. A vertical black line denotes the mean proportion of non-targeting sgRNAs, serving as a reference baseline. (**A–G**) Distributions are shown for 1 bp Ins (**A**), Large Ins (**B**), MH Small Del (**C**), MH Large Del (**D**), NonMH Small Del (**E**), NonMH Large Del (**F**), and Mut (**G**). DSB repair regulators were highlighted and exhibited significant deviations from the overall distributions, confirming that IPGRM can sensitively capture pathway modulators at the sgRNA level.


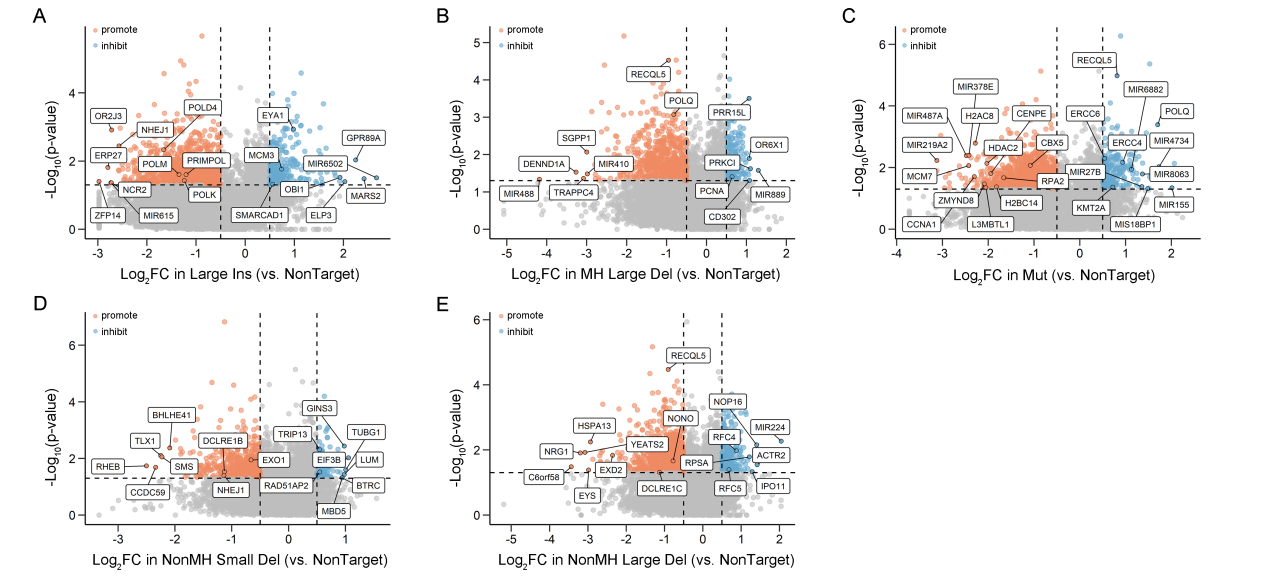


**Supplementary Figure S10.** IPGRM reveals distinct sets of genes regulating specific DSB repair patterns. (**A–E**) Volcano plots showing genome-wide screening results for genes associated with Large Ins (**A**), MH Large Del (**B**), Mut (**C**), NonMH Small Del (**D**), and NonMH Large Del (**E**) repair outcomes. Each point represents a gene or miRNA, with x-axis indicating log₂ fold change (vs. NonTarget controls) and y-axis showing –log₁₀ p values. Orange and blue indicate factors promoting or inhibiting the respective repair types. Representative regulators are labeled. Dashed lines mark significance thresholds.


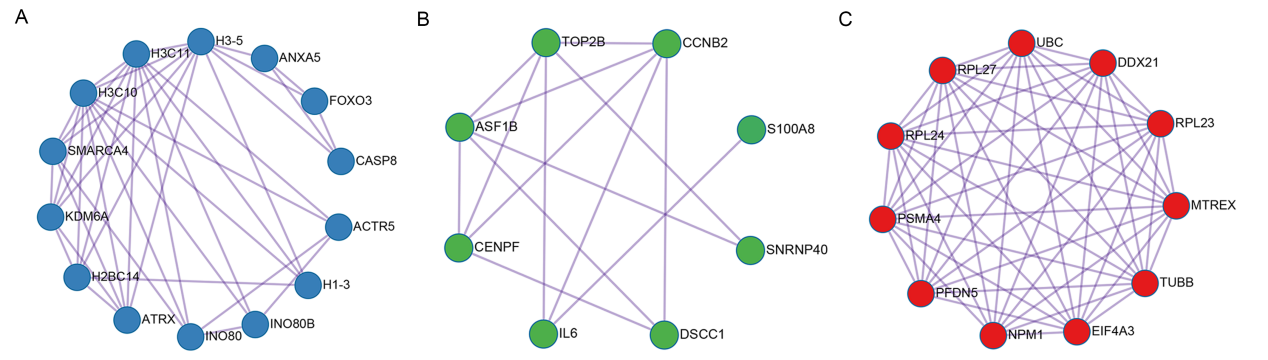


**Supplementary Figure S11.** Protein–protein interaction networks of genes regulating distinct repair outcomes identified by IPGRM. PPI networks constructed from genes significantly associated with 1 bp Ins promotion (**A**) and MH Small Del inhibition (**B** and **C**). Nodes represent individual genes, and edges indicate experimentally validated or high-confidence predicted protein–protein interactions.


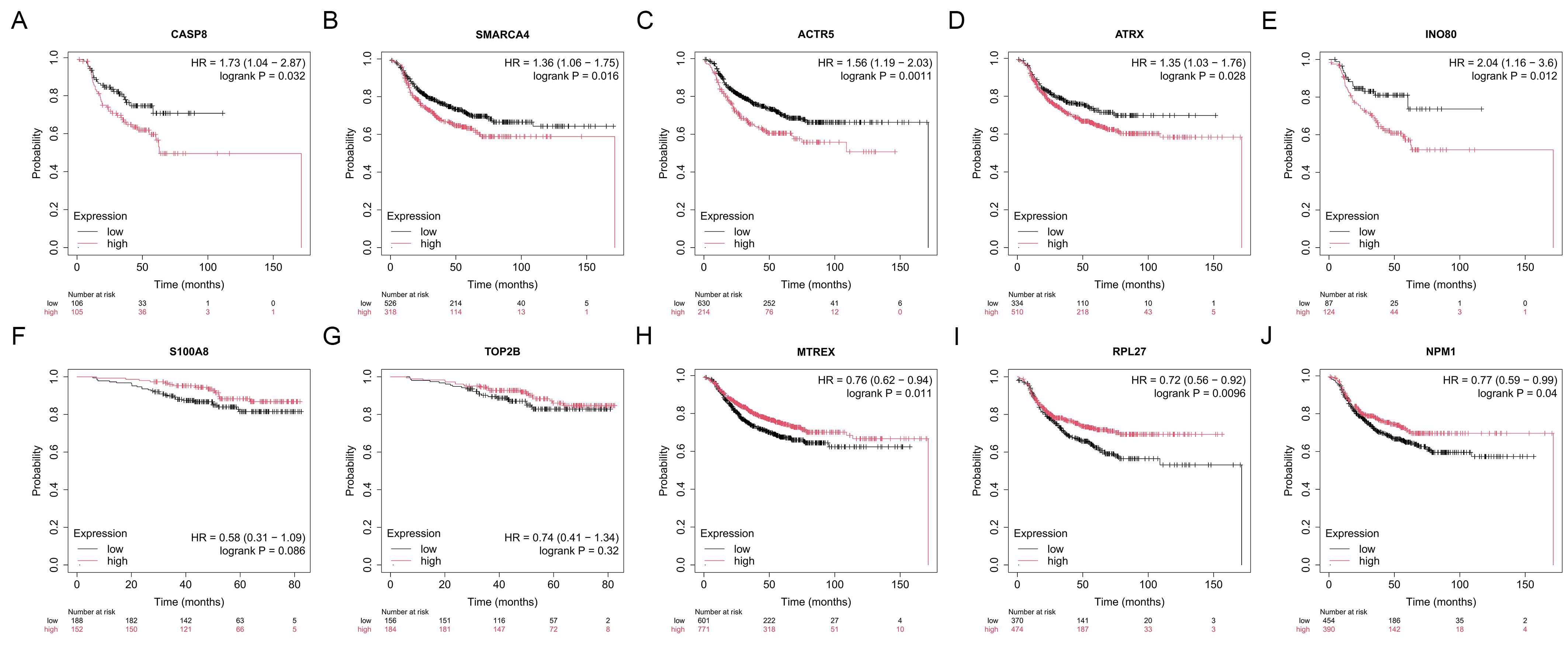


**Supplementary Figure S12.** Survival impact of repair-associated genes in chemotherapy-treated breast cancer. Kaplan–Meier survival curves showing overall survival for breast cancer patients stratified by high (red) and low (black) expression levels of representative genes identified from the IPGRM screen. (**A–E**) Genes promoting 1 bp Ins (CASP8, SMARCA4, ACTR5, ATRX, INO80). (**F–J**) Genes inhibiting MH Small Del (S100A8, TOP2B, MTREX, RPL27, NPM1). Hazard ratios (HR) and log-rank p-values are indicated for each panel.


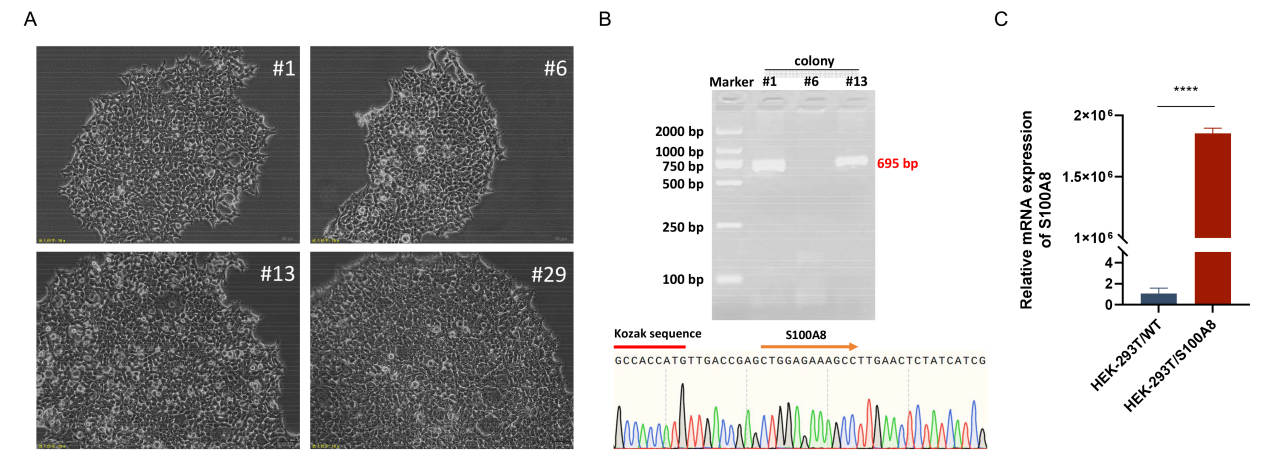


**Supplementary Figure S13.** Establishment and validation of HEK293T cells overexpressing S100A8. (**A**) Representative microscopic images of HEK293T cell colonies (#1, #6, #13, and #29) after stable transfection with the S100A8 overexpression vector. (**B**) Verification of S100A8 integration by colony PCR. A specific band at 695 bp confirmed successful construct insertion; Sanger sequencing validated the Kozak–S100A8 junction sequence. (**C**) Quantification of S100A8 mRNA expression in HEK293T WT and S100A8-OE cells measured by qRT-PCR. n = 3, **** P < 0.0001.


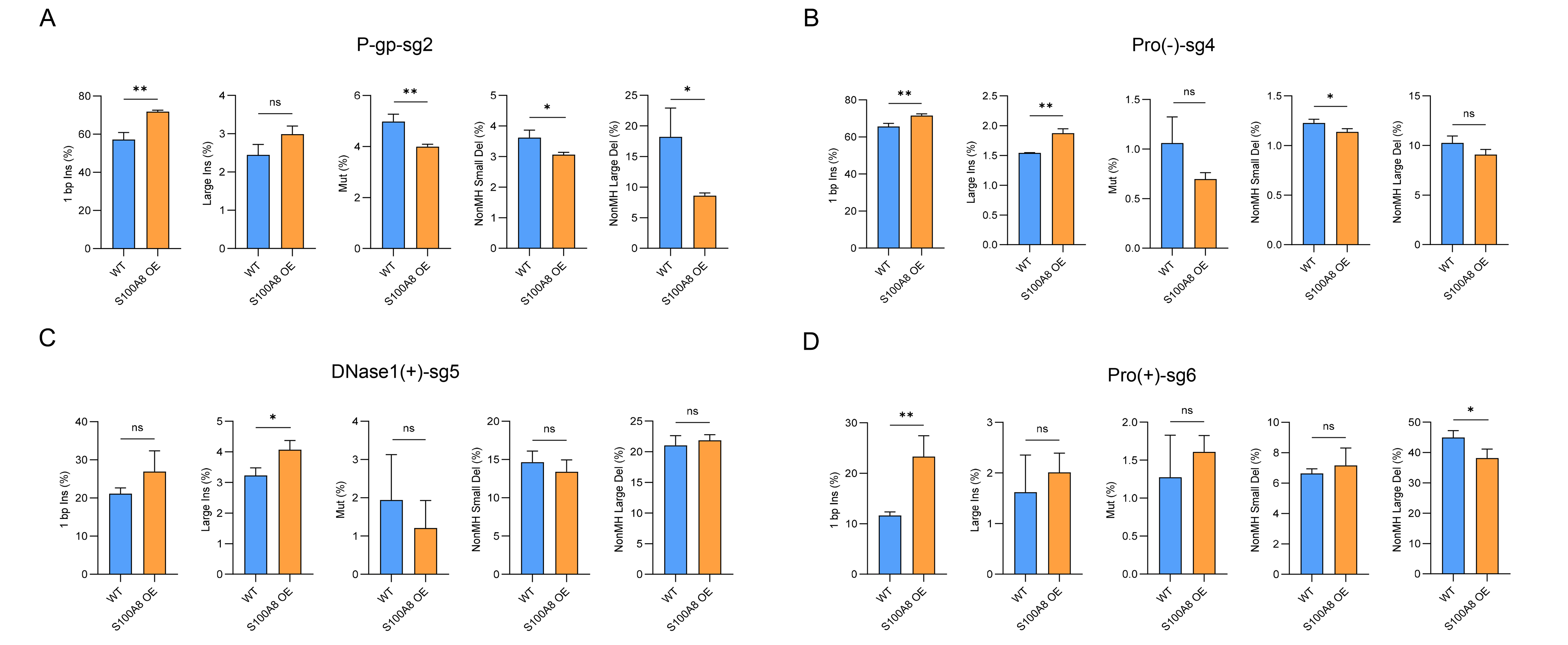


**Supplementary Figure S14.** Broad impact of S100A8 overexpression on diverse repair outcomes. (**A–D**) Quantification of other indel patterns (1 bp Ins, Large Ins, Mut, NonMH Small Del, NonMH Large Del) at four representative target sites in HEK293T WT and S100A8-OE cells. Each bar represents the proportion of the indicated repair type among all edited alleles. n = 3, * P < 0.05; ** P < 0.01; ns, not significant. All values are presented as mean ± SD.


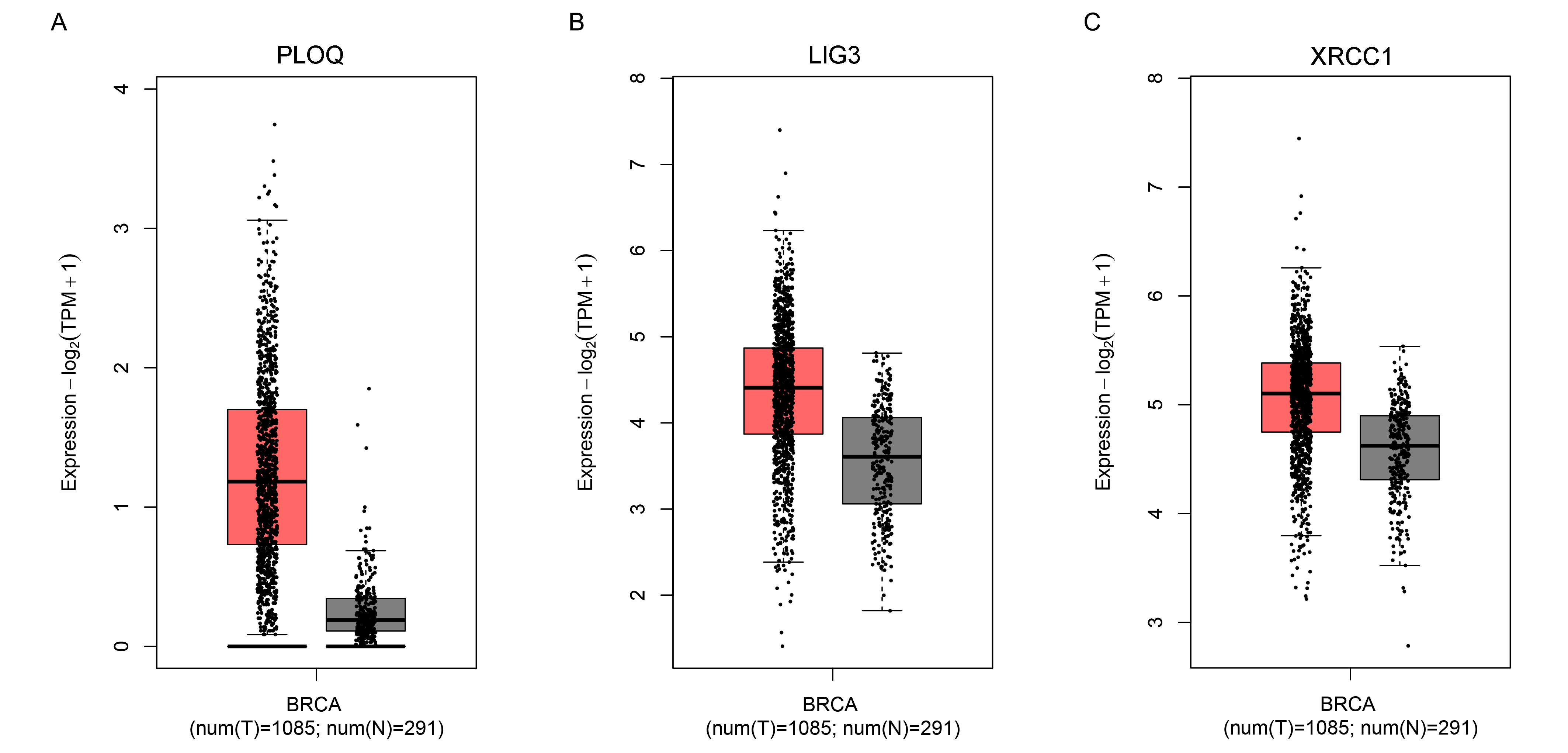


**Supplementary Figure S15.** Expression of MMEJ repair factors in breast cancer (BRCA) based on GEPIA2 dataset. (**A–C**) Gene expression levels of POLQ (**A**), LIG3 (**B**), and XRCC1 (**C**) in breast cancer (BRCA) tumor tissues (T, n = 1085) and normal tissues (N, n = 291). Data were obtained from the GEPIA2 database. All three genes showed elevated expression in tumor samples compared with normal tissues, consistent with their roles in MMEJ repair activity, although the differences did not reach statistical significance.


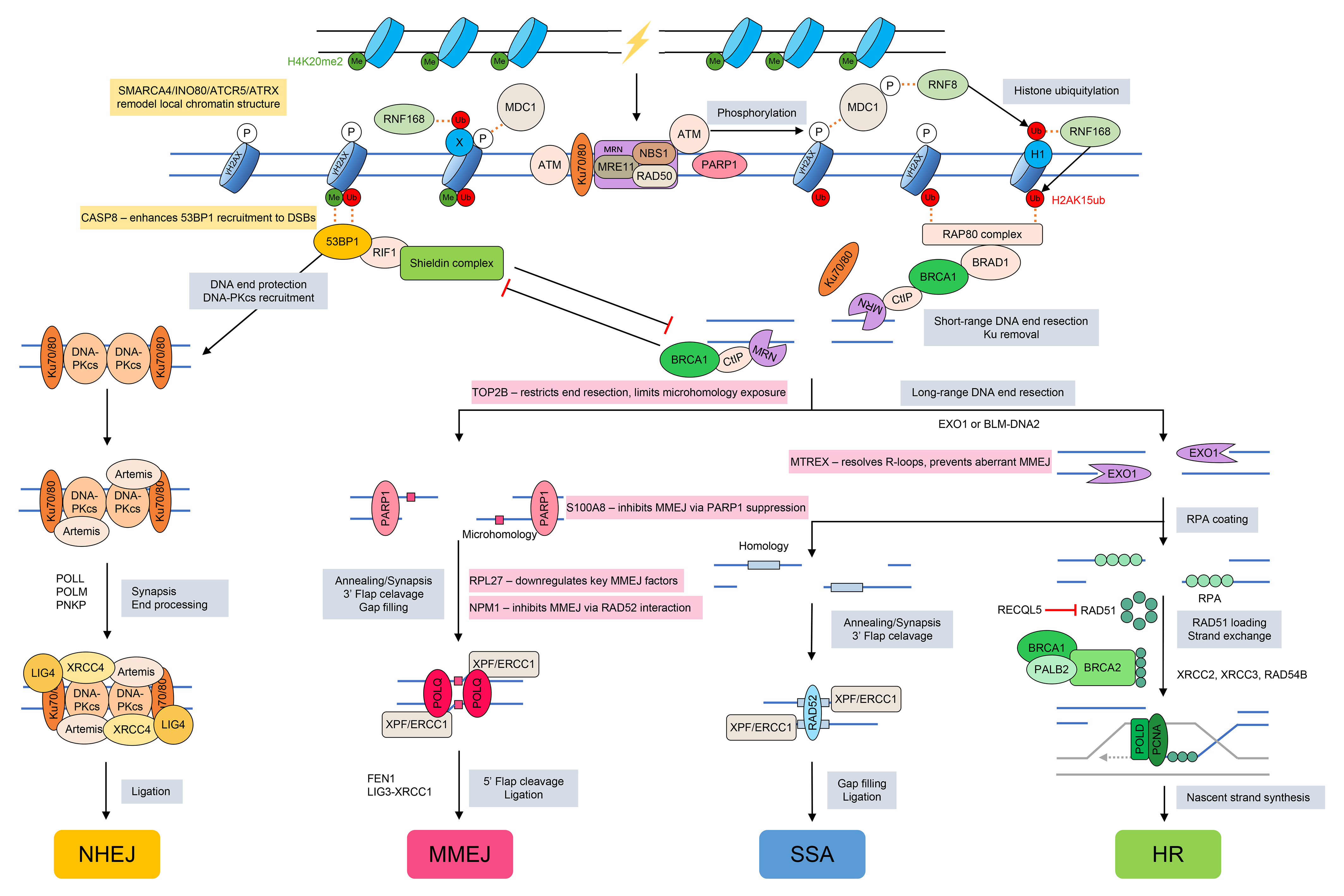


**Supplementary Figure S16.** Proposed model summarizing the regulatory landscape of DSB repair pathways revealed by IPGRM. Chromatin remodelers (SMARCA4, INO80, ATRX, ACTR5) and CASP8 enhance DSB recognition and end protection, while TOP2B and MTREX limit excessive resection. In the MMEJ branch, S100A8, RPL27, and NPM1 suppress repair via PARP1 or RAD52 interference, collectively defining a chromatin-dependent framework that governs pathway choice and repair fidelity.


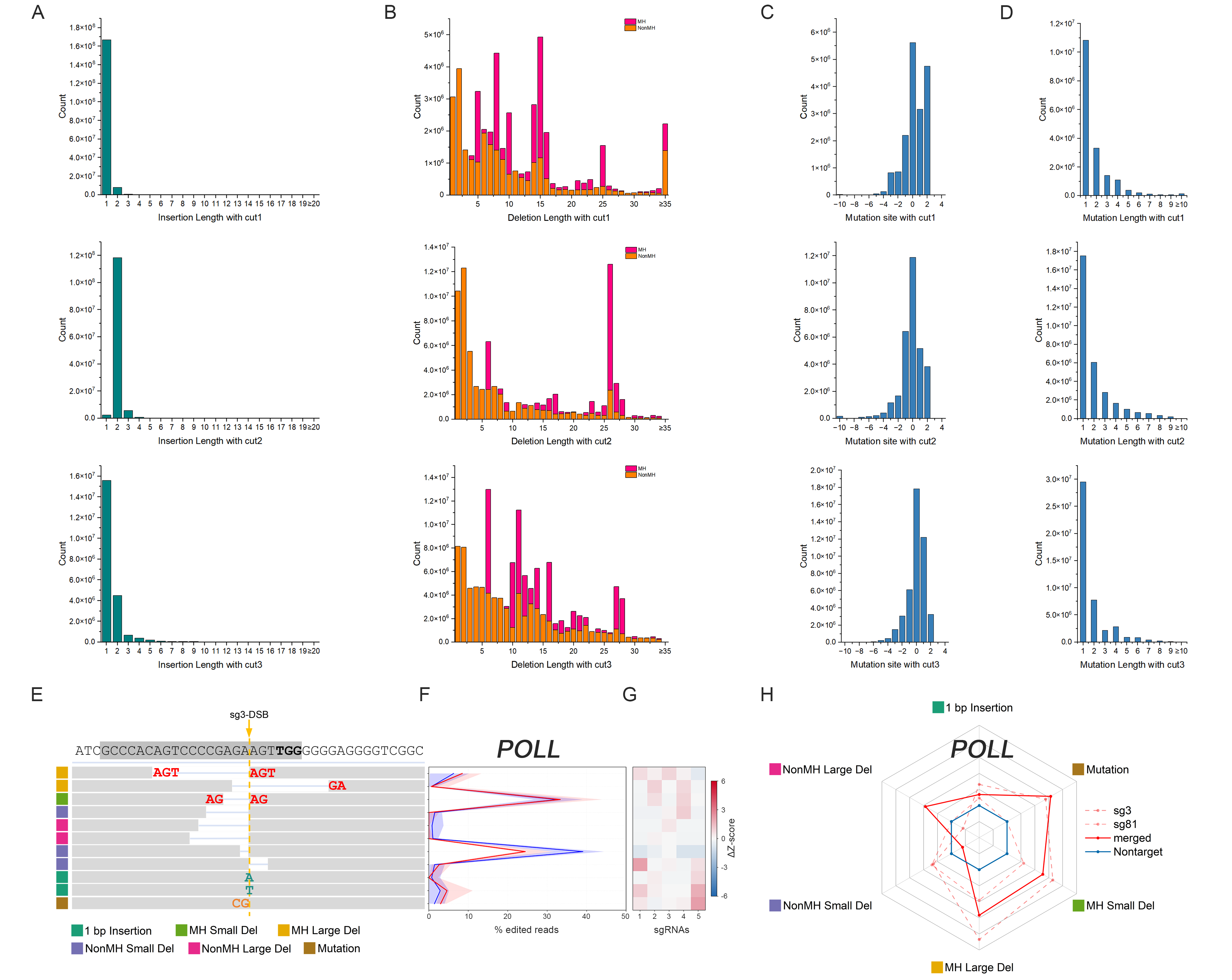


**Supplementary Figure S17.** Cross-study comparison of global repair outcome profiles between IPGRM and REPAIRome. (**A–D**) Global distributions of insertion lengths (**A**), deletion lengths and MH-usage (**B**), and mutation sites (**C**) and lengths (**D**) lengths derived from the REPAIRome dataset, showing dominant 1-bp insertions and enrichment of microhomology-associated deletions, consistent with IPGRM outcome signatures. (**E–H**) Application of the REPAIRome representative-outcome analysis workflow to the IPGRM dataset. Using POLL as an example, outcome profiles (**E–G**) and radar plot summaries (**H**) illustrate increased microhomology-associated deletions upon POLL knockout, producing a directionally interpretable shift in repair outcomes.
